# Genomic characterization of Influenza A (H1N1)pdm09 and SARS-CoV-2 from Influenza Like Illness (ILI) and Severe Acute Respiratory Illness (SARI) cases reported between July–December, 2022

**DOI:** 10.1038/s41598-024-58993-w

**Published:** 2024-05-09

**Authors:** Pushpendra Singh, Kuldeep Sharma, Anudita Bhargava, Sanjay Singh Negi

**Affiliations:** Department of Microbiology, All India Institute of Medical Sciences, Raipur, Chhattisgarh India

**Keywords:** ILI/SARI case, COVID-19, SARS-CoV-2, Influenza A (H1N1)pdm09, Co-infection, SARS-CoV-2, Influenza virus

## Abstract

Influenza Like Illness (ILI) and Severe Acute Respiratory Infection (SARI) cases are more prone to Influenza and SARS-CoV-2 infection. Accordingly, we genetically characterized Influenza and SARS-CoV-2 in 633 ILI and SARI cases by rRT-PCR and WGS. ILI and SARI cases showed H1N1pdm09 prevalence of 20.9% and 23.2% respectively. 135 (21.3%) H1N1pdm09 and 23 (3.6%) H3N2 and 5 coinfection (0.78%) of H1N1pdm09 and SARS-CoV-2 were detected. Phylogenetic analysis revealed H1N1pdm09 resemblance to clade 6B.1A.5a.2 and their genetic relatedness to InfA/Perth/34/2020, InfA/Victoria/88/2020 and InfA/Victoria/2570/2019. Pan 24 HA and 26 NA nonsynonymous mutations and novel HA (G6D, Y7F, Y78H, P212L, G339R, T508K and S523T) and NA (S229A) mutations were observed. S74R, N129D, N156K, S162N, K163Q and S164T alter HA Cb and Sa antibody recognizing site. Similarly, M19T, V13T substitution and multiple mutations in transmembrane and NA head domain drive antigenic drift. SARS-CoV-2 strains genetically characterized to Omicron BA.2.75 lineage containing thirty nonsynonymous spike mutations exhibited enhanced virulence and transmission rates. Coinfection although detected very minimal, the mutational changes in H1N1pdm09 and SARS-CoV-2 virus infected individuals could alter antibody receptor binding sites, allowing the viruses to escape immune response resulting in better adaptability and transmission. Thus continuous genomic surveillance is required to tackle any future outbreak.

## Introduction

Ongoing pandemic of COVID-19 caused by novel Coronavirus SARS-CoV-2 from 2020 to date and an earlier pandemic of Swine flu caused by Influenza A subtype H1N1pdm09 in 2009 (hereafter referred as H1N1pdm09) have evidently shown that the respiratory viruses have been responsible for high mortality and morbidity across the globe^1^. Yearly, around 3–5 million people were reported infected with Influenza virus globally causing 290,000 to 650,000 deaths^2^. As of 17 December 2023, over 772 million confirmed cases of SARS-CoV-2 causing nearly seven million deaths have been reported globally^3^.

Since, both the virus have the similar route of droplets transmission to infect the upper respiratory tract, short incubation period and similar clinical manifestation, there is always the likelihood of these viruses to cause coinfection^1^. Its imperative imminence also evident from various published reports of coinfection^1,4,5^. While H1N1pdm09 emerged as the result of reassortment between Swine, Human and Avian Influenza virus, the origins of SARS-CoV-2 remains unclear, although majority of the reports suggest zoonotic origins from animal sources, varying from Bat to Civet with animal to human transmission not precisely defined^6^. Nevertheless, both these viruses are highly mutation prone and taking the cognizance of the fact, WHO has underlined the importance of continuous periodical monitoring of both these viruses to keep the close watch on any mutational changes in these viruses to initiate appropriate advanced management preparedness including modification in the current vaccine virus^7^. Due to the similarity in clinical presentation of influenza and SARS-CoV-2 infections, the WHO integrated surveillance of both SARS-CoV-2 and Influenza A, B and their subtypes using multiplex reverse transcriptase real time polymerase chain reaction (rRT-PCR) for their detection^7^. WHO categorized the cases into two syndromes Influenza Like Illness (ILI) and Severe Acute Respiratory Illness (SARI) to screen them for detection of Influenza and SARS-CoV-2. Both ILI and SARI cases experience acute respiratory infection with onset of fever ≥ 38 °C and cough for the last 10 days, with SARI cases including additional parameters requiring hospitalization^7^.

In India, very few data of screening of ILI/SARI cases has been published checking prevalence of H1N1pdm09 and SARS-CoV-2. An earlier case report has found coinfection of 0.04% from Western, Northern and Southern India^1^. Even these cases were not reported for genomic characterization of mutational pattern. Further, there is no published data from the state of Chhattisgarh, Central India.

The objective of the current study was genetic characterization of SARS-CoV-2 and H1N1pdm09 viruses and investigation of their patterns of co-infection in ILI and SARI cases by rRT-PCR, whole genome sequencing (WGS) and bioinformatics analyses for strain identification, genetic relatedness and mutational patterns analyses to describe their possible effect on viral proteins.

## Materials and methods

### Samples collection

State-Level Virus Research and Diagnostic Laboratory (VRDL), All India Institute of Medical Sciences (AIIMS), Raipur, a government designated state nodal diagnostic and treatment center for SARS-CoV-2/COVID-19 and Influenza surveillance under “ILI/SARI Sentinel Surveillance: A pan-India project” obtained the institutional ethical approval for the current study (AIIMSRPR/IEC/2021/1044). A total of six hundred thirty three (633) ILI/SARI cases visiting AIIMS, Raipur and various district hospitals of Chhattisgarh between 1st July to 30th December, 2022 were included in the study. The combined clinical sample of nasopharyngeal (NPS) and oropharyngeal swabs (OPS) were collected in viral transport media (VTM) after obtaining informed consent from all subjects and/or their legal guardian(s). The combined NPS and OPS samples were processed for following investigation in accordance with the relevant guidelines and regulations.

### rRT-PCR for Influenza and COVID-19 diagnosis and subtyping

The viral RNA extraction was performed from the collected clinical samples using commercially available QIAamp Viral RNA Mini Kit (Cat. No. 52904) as per the manufacturer’s instructions. The extracted RNA was used to perform rRT-PCR assay developed and provided by National Institute of Virology (NIV), Pune, India for simultaneous detection of SARS-CoV-2, Infleunza A and its subtype (H1N1pdm09 and H3N2) and Influenza B and its subtype (Victoria or Yamagata) by using respective primers and probes^1,8^.

### Whole genome sequencing

A total of thirty nine (39) randomly selected positive samples from 17 ILI and 22 SARI cases including five coinfection (01 ILI and 04 SARI cases) were processed for the respective viral WGS. The cDNA library has been prepared using extracted RNA and Superscript III OneStep RT-PCR (ThermoFisher Scientific, USA) along with hexamer random primer at 25 °C for 10 min, 42 °C for 50 min and 70 °C for 15 min. Second strand synthesis master mix was subsequently added and kept at 16 °C for 60 min to synthesize the second strand. AMPure XP beads were utilized to purify the complete double-stranded DNA^9^. Nextera DNA Flex Library Prep Kit (Illumina, USA) was used for library preparation according to the manufacturer instructions. Briefly, the DNA was tagmented with bead-linked transposomes, followed by eight cycles of amplification of the tagmented DNA with enhanced PCR mix and index adapters. The prepared libraries were then cleaned using 0.9 × sample purification beads and quantified by the Qubit dsDNA High Sensitivity assay kit (Invitrogen) followed by sequencing on the MiniSeq platform (Illumina) using paired-end Mid-output Cartridge (300 cycle)^10,11^.

### Data assembly and submission

FastQ sequence raw data obtained for all 39 samples in MiniSeq local run manager (Illumina, USA) were processed in the sequential manner for trimming to remove indexes adapter, subsequent mapping with reference sequence and final assembly into consensus sequences using QIAGEN CLC Genomics Workbench version 21.0.4. The Influenza A/California/07/2009 (H1N1), (FJ984386, FJ969827, FJ969529–FJ969531, FJ969536, FJ969538, and FJ969540), and SARS-CoV-2 Wuhan strain NC045512 were used as reference sequence. The thirty nine whole genome consensus sequences of influenza H1N1pdm09 and 5 sequences of SARS-CoV-2 were submitted to the National Center for Biotechnology Information (NCBI), and their details and accession numbers are mentioned in Supplementary Table 1.

### Phylogenetic analysis and variant identification

For phylogenetic analysis and variant identification, our thirty nine sequences of H1N1pdm09 and 5 sequences of SARS-CoV-2 were aligned with forty sequences of the Influenza A various reference clades and the representative sequences of SARS-CoV-2 using the MAFFT (multiple sequence alignment based on fast Fourier transform)^12^. Clade defining sequences were indicated by Next strain platform (https://nextstrain.org/flu/seasonal). The phylogenetic tree was constructed using the Neighbour-joining (NJ) method with a bootstrap resampling (1000 replicates) protocol by the MEGA-X bioinformatics tool version 10.2.5^13^. The nucleotide substitutions/deletions and subsequent amino acid (aa) change in the sequences were identified with the help of a reference prototype to explore their effect on the protein stability, structure and function.

### Structure modelling and evaluation

The 3D modelling of hemagglutinin (HA) and neuraminidase (NA) gene of the reference H1N1pdm09 sequence was used to study the effect of specific aa mutational changes in the HA and NA gene from the thirty nine sequences. The homology model of HA and NA protein was obtained through the SWISS-MODEL server using template structure (PDB ID: 3UBE for HA and 5NWE for NA). Further, the 3D refine server was used to restrain the model by using the energy minimization process as it effectively escapes any local minima and moves closer to the native protein structure^14^. After optimization, the stereochemical quality of the obtained model was validated by using the various assessment tools, namely Ramachandran plot and G-factor in PROCHECK^15^, ERRAT Plot^16^ and ProSA Local energy profile^17^ programs available freely from the Structural Analysis and Verification Server (SAVES) (http://nihserver.mbi.ucla.edu/SAVES). Subsequently, the modelled structure was imported into the Schrödinger software, and in it, the various mutations of substitution and deletion identified in our sequences were added. The visualization of all the predicted models was done in PyMOL to analyse the mutations and images output.

### Ethics statement

ILI/SARI surveillance under “ILI/SARI Sentinel Surveillance: A pan-India project” has obtained the institutional ethical approval (AIIMSRPR/IEC/2021/1044).

## Results

**Figure 1:**
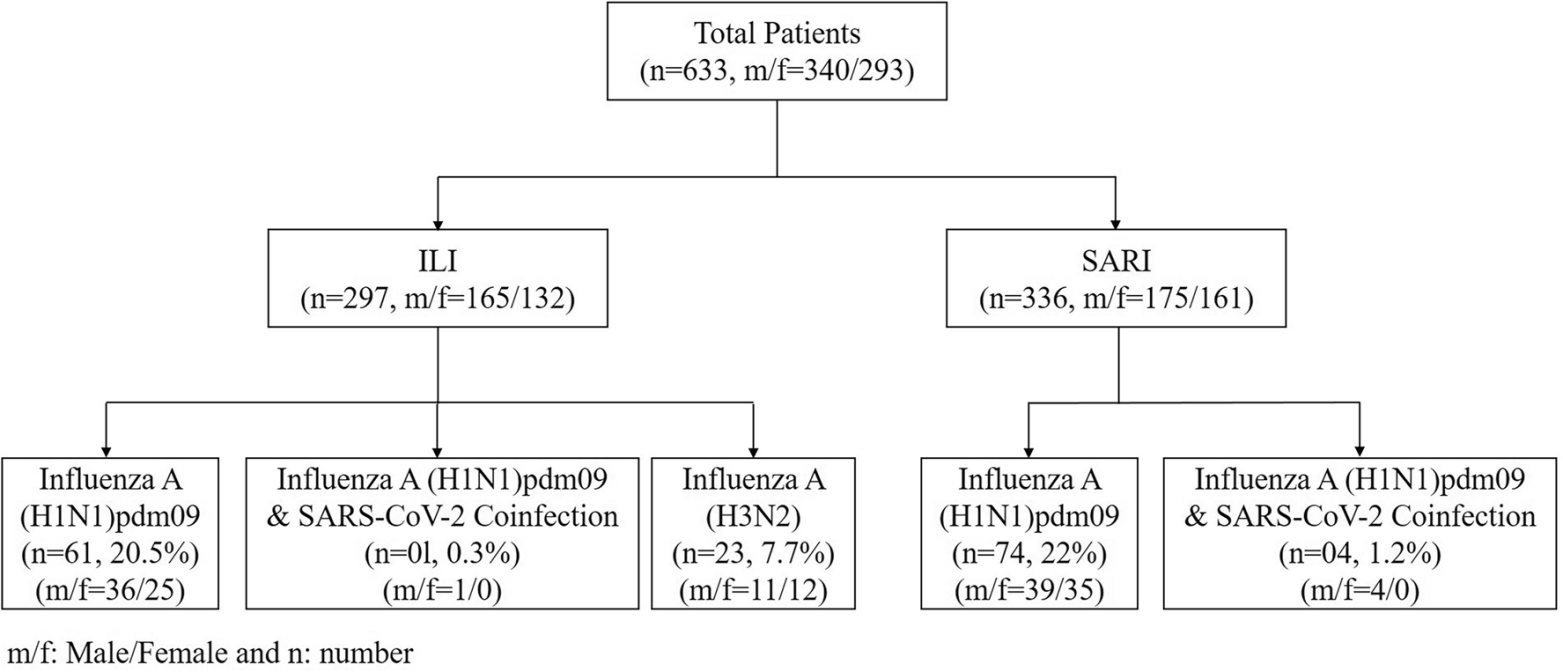
Patients diagnosed for Influenza A, B and their subtypes and SARS-CoV-2.

A total of 297 ILI and 336 SARI cases were screened for the presence or absence of Influenza A and its subtype H1N1pdm09 and H3N2, Influenza B and its subtype Victoria or Yamagata and SARS-CoV-2 by multiplex rRT-PCR. Gender distribution revealed 53.71% male (n = 340) and 46.28% female (n = 293), with a median age of 38.13 years. Among 297 ILI cases, H1N1pdm09 was detected in 61 cases (20.5%), H3N2 in 23 cases (7.7%) and co-infection of H1N1pdm09 and SARS-CoV-2 in one case(0.3%) (Fig. 1). Among these ILI patients males were found more affected than females, although the difference was not found significant either in H1N1pdm09 (χ^2^ = 0.372, p = 0.541, p > 0.05) or H3N2 (χ^2^ = 0.603, p = 0.437, p > 0.05). On the other hand in 336 SARI cases, H1N1pdm09 was detected in 74 cases (22%), co-infection in four (04) cases (1.2%) and no H3N2 infection (Fig. 1). In SARI cases too, males were found more affected than females, however the difference was not found statistically significant in (χ^2^ = 0.377, p = 0.539, p > 0.05). Influenza B was not detected in any of the ILI or SARI cases. Thus, overall 163 Influenza A comprising 140 H1N1pdm09 (62 ILI and 78 SARI) and 23 H3N2 (all ILI) cases were detected. These 140 H1N1pdm09 included 05 cases of coinfection with SARS-CoV-2 infection also. In both ILI/SARI, Influenza A was found affecting more males (n = 91, 55.8%) than females (n = 72, 44.4%), although difference was not found statistically significant (χ^2^ = 0.665, p = 0.414, p > 0.05). The average mean age affected was 38.66 Yrs. H1N1pdm09 infection was observed more in elderly patients while H3N2 and co-infection was seen more seen in younger patients (median age 21–23 years) (data not shown).

Analysis of clinical symptoms revealed that fever and cough was the predominant symptom in all cases followed by sore throat (40.3%), sputum production (36.8%), nausea (33.2%), chest pain (27.2%) and other feature (Table 1). Categorical analysis of ILI/SARI cases revealed clinical features more manifested in SARI cases. Five patients, who presented with co-infection of Influenza A H1N1pdm09 with SARS-CoV-2 were found vaccinated against SARS-CoV-2 and unvaccinated against Influenza A H1N1pdm09. Among these coinfection patients, one 55 year old male SARI case has succumbed due to H1N1pdm09 Pneumonia for 5 days and ARDS for one day.

**Table 1:** Characteristics symptoms of ILI/SARI cases and laboratory-confirmed influenza A H1N1 and H3N2 positive cases.

Symptoms/patient infection category	SARI/ILI cases (633) % (N)	H1N1 positive cases including coinfection (N = 140)		H3N2 positive cases (all ILI) (N = 23) % (N)
		ILI (62) % (N)	SARI (78) % (N)	
Fever	100 (633)	100 (62)	100 (78)	100 (23)
Cough	100 (633)	100 (62)	100 (78)	100 (23)
Sputum production	36.8 (233)	27.4 (17)	33.3 (26)	43.5 (10)
Sore throat	40.3 (255)	40.3 (25)	42.3 (33)	34.8 (8)
Nausea	33.2 (210)	37.1 (23)	32.1 (25)	43.5 (10)
Chest pain	27.2 (172)	17.7 (11)	30.7 (24)	26.1 (6)
Haemoptysis	19.7 (125)	16.1 (10)	24.3 (19)	17.4 (4)
Diarrhea	11.7 (74)	8.1 (5)	15.3 (12)	8.7 (2)
Malaise/fatigue	10 (63)	6.4 (4)	7.6 (6)	8.7 (2)
Breathing difficulty	10.9 (69)	3.2 (2)	10.2 (8)	0 (0)

**Figure 2:**
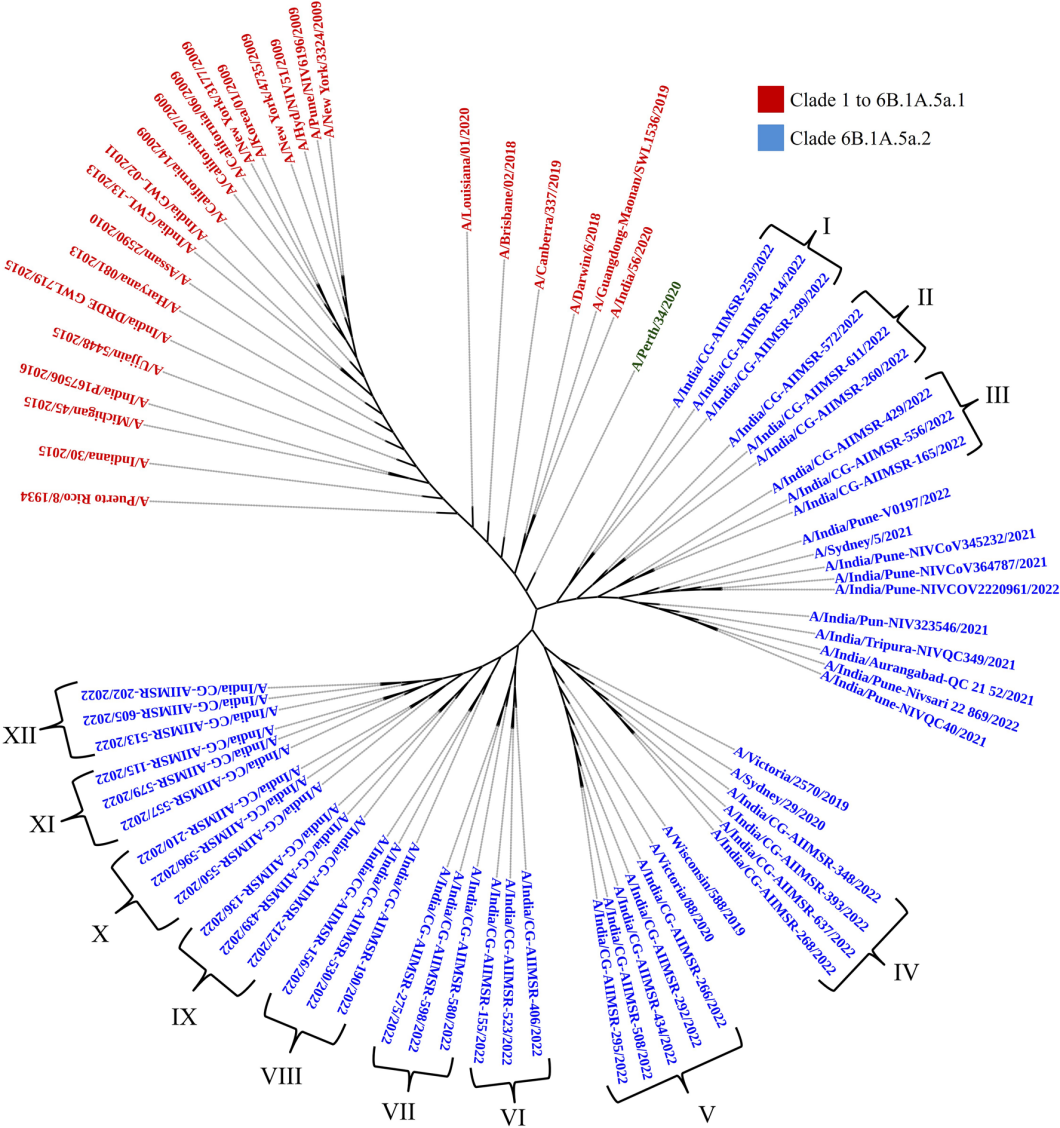
Phylogenetic analysis of HA gene sequences.

**Figure 3:**
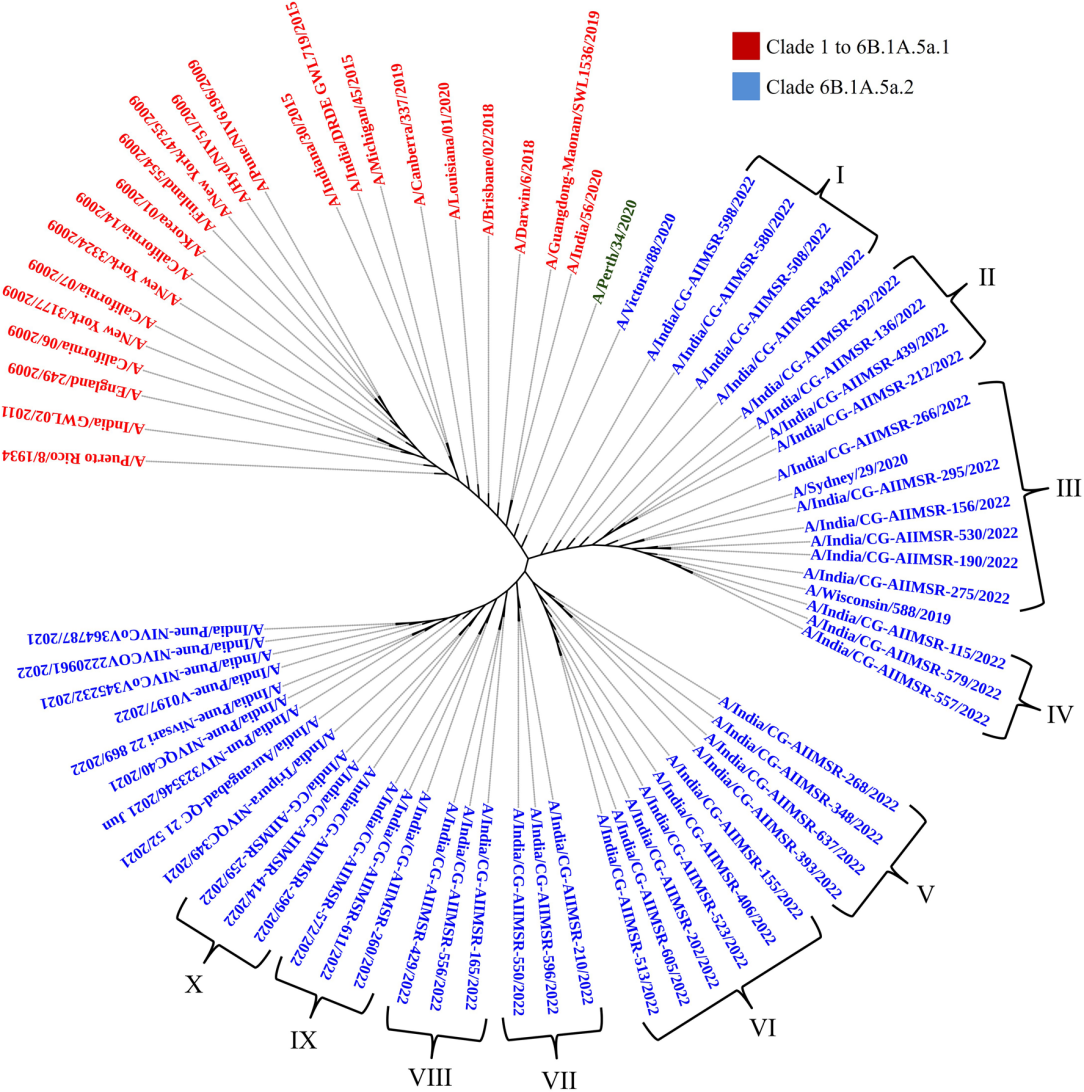
Phylogenetic analysis of NA gene sequences.

Phylogenetic analysis of the HA and NA gene sequences from all thirty nine isolates of (H1N1)pdm09 together with biologically diverse contemporaneous global Influenza A (H1N1)pdm09 viral sequences, including sequences recovered in India in previous years revealed that all sequences belonged to clade 6B.1A.5a2. All Influenza A H1N1 strains were genetically related to A/Perth/34/2020, A/Victoria/2570/2019 and A/Victoria/88/2020 strains (Figs. 2 and 3). In the phylogenetic analysis of HA sequences, 12 subclusters (I–XII) were observed, while in NA they observed as ten clusters (I–X) of virus strains. Subcluster genetic closeness was variable: subcluster III was phylogenetically related to A/Sydney/29/2020 while subcluster IV was phylogenetically related to A/Wilconsin/588/2019.

### Mutational analysis of HA

A total of twenty four aa non-synonymous pan signature mutations were observed in all analyzed HA gene. These noted mutations were S74R, S84N, D97N, N129D, K130N, N156K, L161I, S162N, K163Q, S164T, S183P, S185I, S203T, I216T, V250A, A256T, N260D, K283E, I295V, I321V, E374K, S451N, E499K, and E506D. Some mutations other than these pan mutations were also observed in HA gene of few H1N1pdm09 strains with variable frequencies in Table 2. Notable mutations Y78H, A186T, Q189E, E224A, T508K and S523T forming group III and G6D, Y7F, P83S, P212L, G339R forming group IX were only observed in SARI cases while P83S, I96R and C538F representing group VII were observed confined to ILI cases on phylogenetic analysis. Rest of the groups were observed to have both the ILI and SARI cases with specific mutations present in both clinical conditions (Table 2).

**Table 2:** Mutational aa changes other than the pan non-synonymous mutation in the HA protein of Influenza A (H1N1)pdm09.

S. no.	Group	Isolates	Clinical condition	Mutations
	I.	A/India/CG-AIIMSR-259/2022 A/India/CG-AIIMSR-414/2022 A/India/CG-AIIMSR-299/2022	ILI	V30D, P83S, H273Q
			ILI	
			SARI	
	II.	A/India/CG-AIIMSR-572/2022 A/India/CG-AIIMSR-611/2022 A/India/CG-AIIMSR-260/2022	SARI	None other than pan mutations
			ILI	
			ILI	
	III.	A/India/CG-AIIMSR-429/2022 A/India/CG-AIIMSR-556/2022 A/India/CG-AIIMSR-165/2022	SARI	Y78H, A186T, Q189E, E224A,T508K, S523T
			SARI	
			SARI	
	IV	A/India/CG-AIIMSR-348/2022 A/India/CG-AIIMSR-393/2022 A/India/CG-AIIMSR-637/2022 A/India/CG-AIIMSR-268/2022	ILI	P83S
			ILI	
			SARI	
			SARI	
	V	A/India/CG-AIIMSR-266/2022 A/India/CG-AIIMSR-292/2022 A/India/CG-AIIMSR-434/2022 A/India/CG-AIIMSR-508/2022 A/India/CG-AIIMSR-295/2022	SARI	P83S
			SARI	
			ILI	
			SARI	
			SARI	
	VI	A/India/CG-AIIMSR-406/2022 A/India/CG-AIIMSR-523/2022 A/India/CG-AIIMSR-155/2022	SARI	P83S, P297L, V503E, L505M, C538F
			ILI	
			SARI	
	VII	A/India/CG-AIIMSR-580/2022 A/India/CG-AIIMSR-598/2022 A/India/CG-AIIMSR-275/2022	ILI	P83S, I96R, C538F
			ILI	
			ILI	
	VIII	A/India/CG-AIIMSR-190/2022 A/India/CG-AIIMSR-530/2022 A/India/CG-AIIMSR-156/2022	SARI	P83S, V361F, D364N, G463C
			ILI	
			SARI	
	IX	A/India/CG-AIIMSR-212/2022 A/India/CG-AIIMSR-439/2022 A/India/CG-AIIMSR-136/2022	SARI	G6D, Y7F, P83S, P212L, G339R
			SARI	
			SARI	
	X	A/India/CG-AIIMSR-550/2022 A/India/CG-AIIMSR-596/2022 A/India/CG-AIIMSR-210/2022	ILI	P83S, F111Y, P282G, A362G, A363P
			ILI	
			SARI	
	XI	A/India/CG-AIIMSR-557/2022 A/India/CG-AIIMSR-579/2022 A/India/CG-AIIMSR-115/2022	ILI	P83S, L329Q, V525A, V528I
			ILI	
			SARI	
	XII	A/India/CG-AIIMSR-513/2022 A/India/CG-AIIMSR-605/2022 A/India/CG-AIIMSR-202/2022	ILI	P83S, P304Q, T368K, C475Y, V525A, F535L
			SARI	
			SARI	

### Mutational analysis of NA

A total of 26 non-synonymous pan signature mutations were observed. These mutations included V13I, M19T, I34V, L40I, N44S, Q51K, Y66F, F74S, G77R, V81A, I188T, N200S, N222K, V241I, N248D, V264I, N270K, I314M, I321V, N369K, N386K, I389K, D416N, K432E, N449D, T452I. Apart from these pan mutations, other non-synonymous mutations specific to certain strains clusters were mentioned in Table 3. Notable sole mutation S299A was found confined only in SARI cases forming group II on phylogenetic analysis. All other group specific mutations were present in both ILI and SARI cases (Table 3).

**Table 3:** Neuraminidase aa mutation other than pan mutations and specific to certain viral strains cluster.

S. no	Group	Isolates	Clinical condition	Mutations
	I.	A/India/CG-AIIMSR-598/2022 A/India/CG-AIIMSR-434/2022 A/India/CG-AIIMSR-508/2022 A/India/CG-AIIMSR-580/2022	ILI	No other mutation detected
			ILI	
			SARI	
			ILI	
	II.	A/India/CG-AIIMSR-292/2022 A/India/CG-AIIMSR-212/2022 A/India/CG-AIIMSR-439/2022 A/India/CG-AIIMSR-136/2022	SARI	S299A
			SARI	
			SARI	
			SARI	
	III.	A/India/CG-AIIMSR-266/2022 A/India/CG-AIIMSR-275/2022 A/India/CG-AIIMSR-190/2022 A/India/CG-AIIMSR-530/2022 A/India/CG-AIIMSR-156/2022 A/India/CG-AIIMSR-295/2022	SARI	No other mutation detected
			ILI	
			SARI	
			ILI	
			SARI	
			SARI	
	IV.	A/India/CG-AIIMSR-557/2022 A/India/CG-AIIMSR-579/2022 A/India/CG-AIIMSR-115/2022	ILI	Y170H, R460H
			ILI	
			SARI	
	V	A/India/CG-AIIMSR-348/2022 A/India/CG-AIIMSR-393/2022 A/India/CG-AIIMSR-637/2022 A/India/CG-AIIMSR-268/2022	ILI	R107S, S110A, F121I
			ILI	
			SARI	
			SARI	
	VI	A/India/CG-AIIMSR-406/2022 A/India/CG-AIIMSR-523/2022 A/India/CG-AIIMSR-155/2022 A/India/CG-AIIMSR-513/2022 A/India/CG-AIIMSR-605/2022 A/India/CG-AIIMSR-202/2022	SARI	Y100N, F121I
			ILI	
			SARI	
			ILI	
			SARI	
			SARI	
	VII	A/India/CG-AIIMSR-550/2022 A/India/CG-AIIMSR-596/2022 A/India/CG-AIIMSR-210/2022	ILI	I17T
			ILI	
			SARI	
	VIII	A/India/CG-AIIMSR-429/2022 A/India/CG-AIIMSR-556/2022 A/India/CG-AIIMSR-165/2022	SARI	I17T, N309D
			SARI	
			SARI	
	IX	A/India/CG-AIIMSR-260/2022 A/India/CG-AIIMSR-572/2022 A/India/CG-AIIMSR-611/2022	ILI	S266L
			SARI	
			ILI	
	X	A/India/CG-AIIMSR-259/2022 A/India/CG-AIIMSR-414/2022 A/India/CG-AIIMSR-299/2022	ILI	H297Q
			ILI	
			SARI	

**Figure 4:**
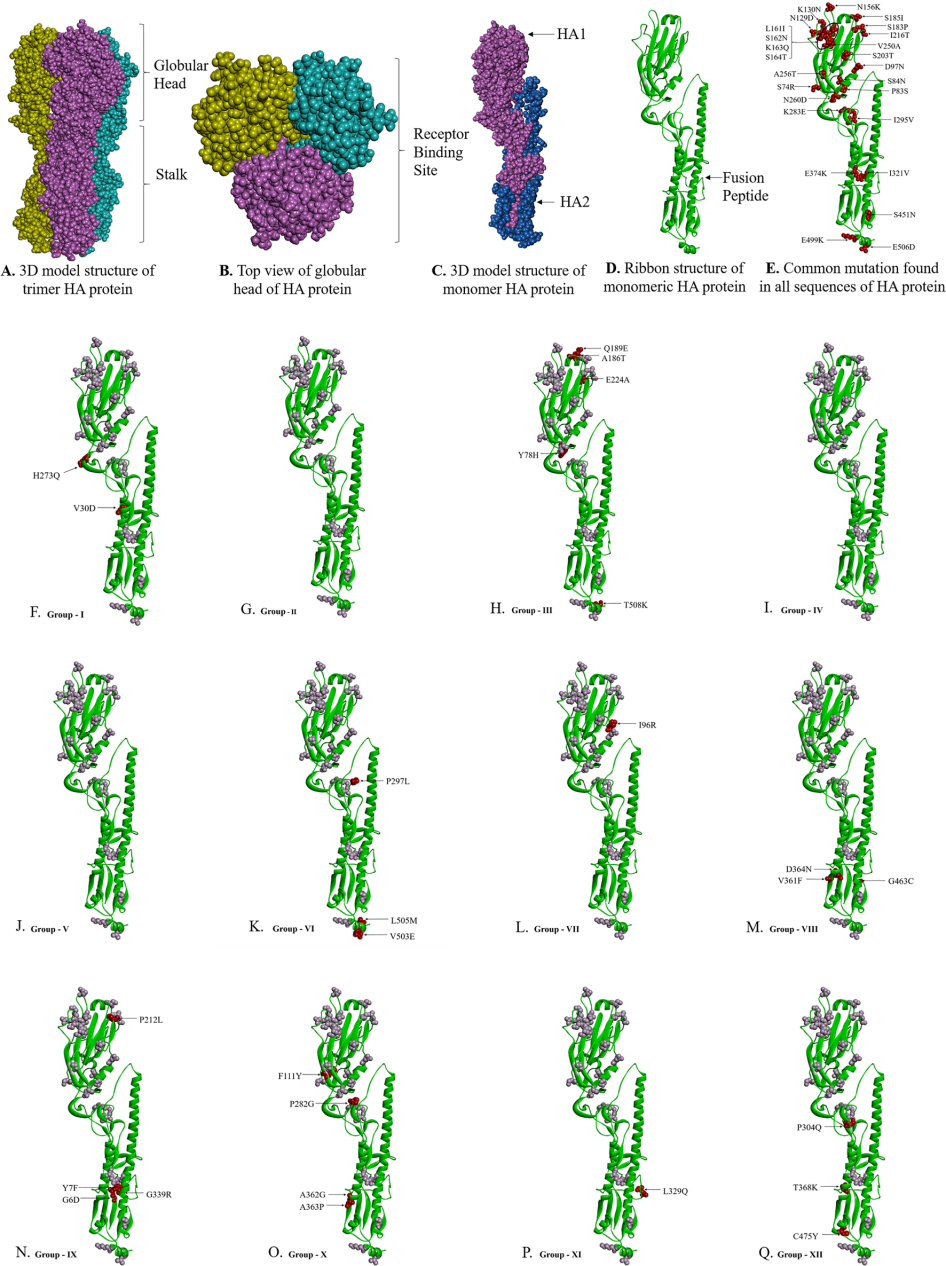
Three-dimensional (3D) structure of HA protein. (**A**) front view trimeric HA protein, (**B**) top view (receptor binding domain) trimeric HA protein, (**C**) monomeric HA, (**D**) ribbon structure of monomeric HA protein, (**E**) common mutation (mutated residue highlighted in red colour) found in all sequences of HA protein, and (**F**–**Q**). Group-wise specific mutations (red) other than common mutation (light grey) in the HA protein.

**Figure 5:**
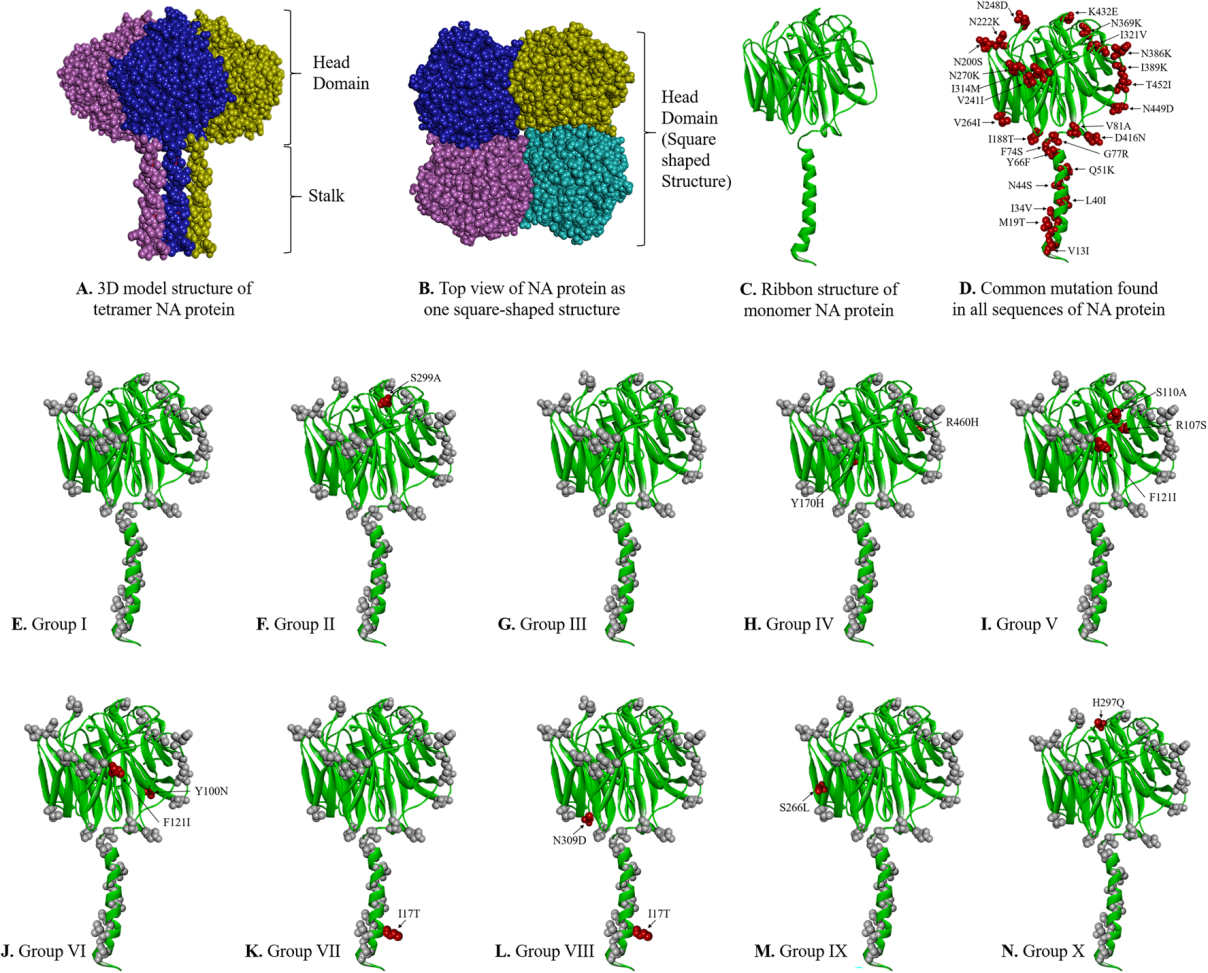
Three-dimensional (3D) structure of NA protein. (**A**) front view trimeric NA protein, (**B**) top view (receptor binding domain) trimeric NA protein, (**C**) ribbon structure of monomeric NA, D. NA protein showing common mutation (mutated residue highlighted in red colour) found in all sequences of NA protein, and (**E**–**N**) Group-wise specific mutations (red) other than common mutation (light grey) in the NA protein.

Three dimensional structural analysis of HA and NA protein for our 39 strains were also performed (Figs. 4, 5). All the mutations in HA and NA were represented in their monomeric protein ribbon structure (Figs. 4E, 5D). All the pan mutations in HA and NA were represented in light green colour while mutation specific to various cluster groups were represented in red colour (Figs. 4F–Q, 5E–N).

**Figure 6:**
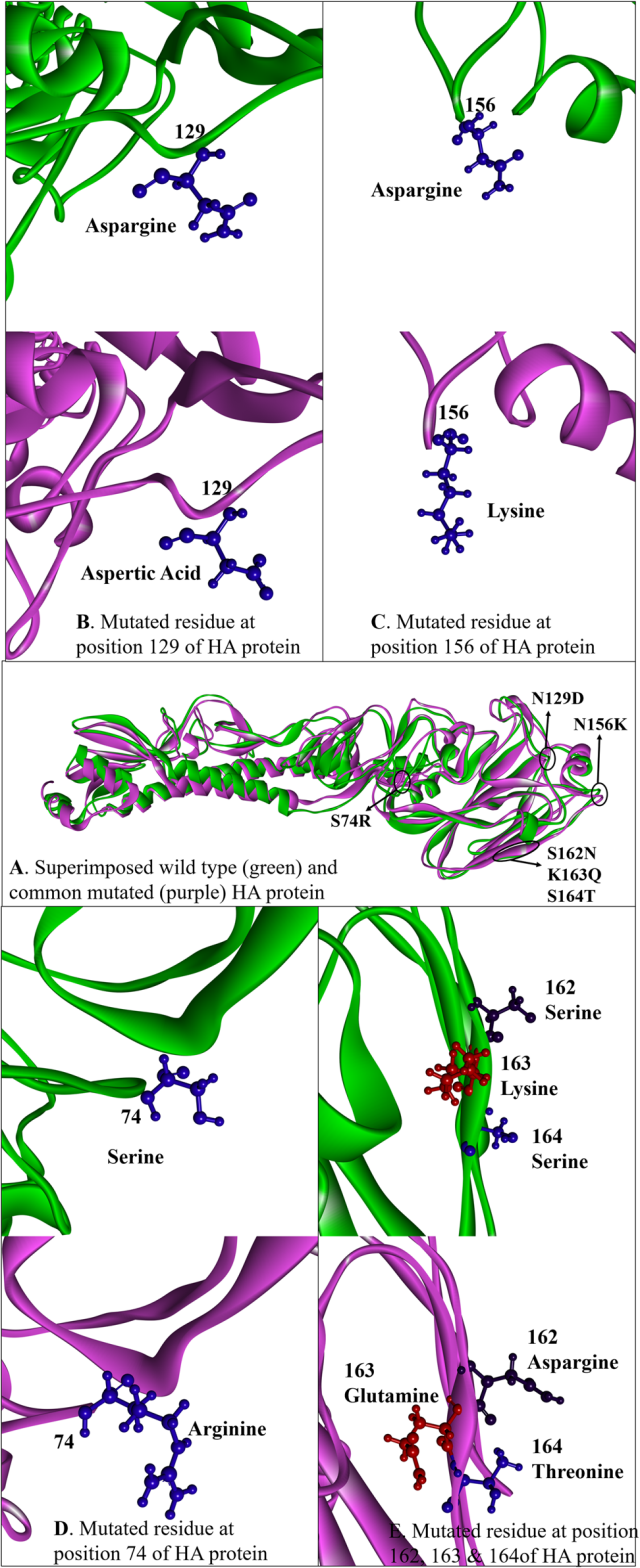
(**A**) Superimposed structures of reference influenza A (H1N1) 2009 pandemic, PDB: 3UBE and mutated HA, where reference structure is highlighted in green and mutated structure in magenta. (**B**–**E**) Substitutions at the binding site residues in receptor binding domain are highlighted in blue.

Antibody receptor binding site in HA protein were further analyzed vis-a-vis reference H1N1pdm09 sequence with respect to their PDB structure (Fig. 6). The mutation of S74R, N129D, N156K, S162N, K163Q and S164T appears to significantly changed Sa and Sb antibody recognizing sites helping in better adaptability of the virus for more efficient human transmission.

**Figure 7:**
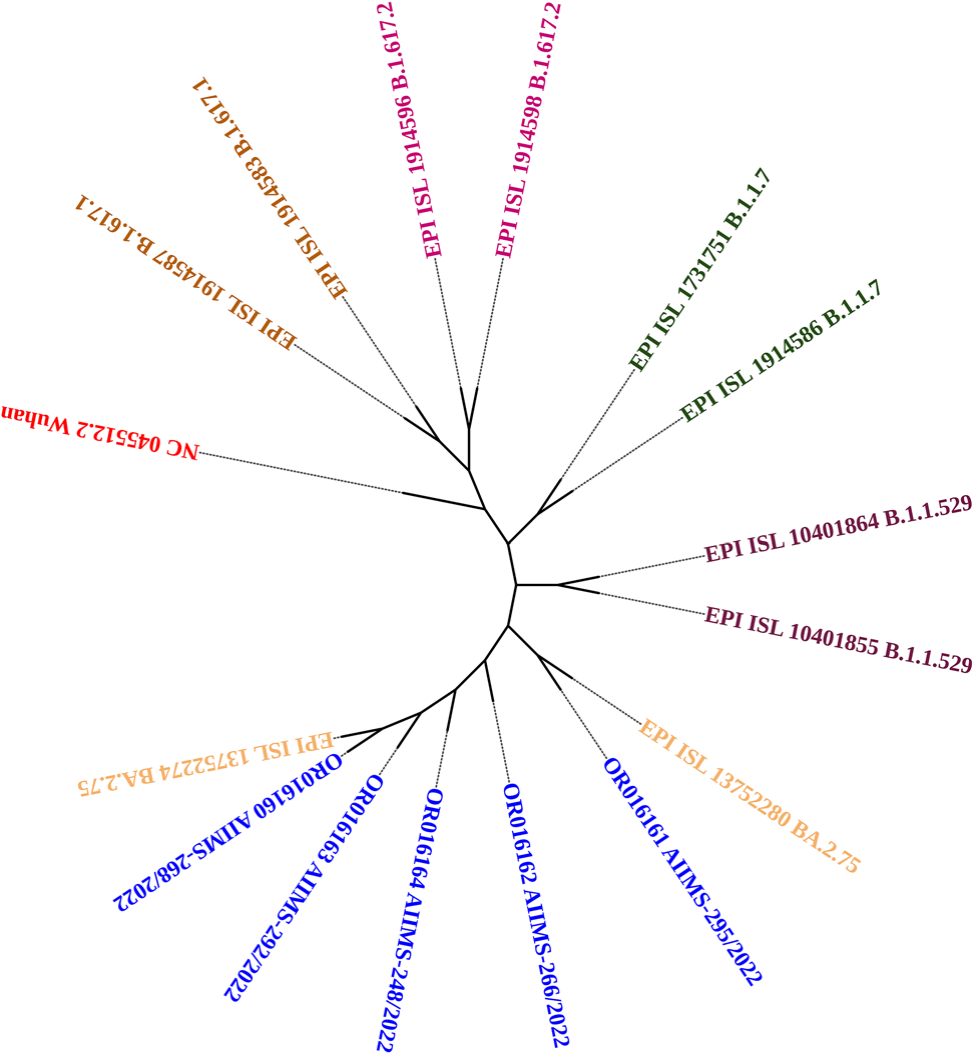
Phylogenetic analysis of five SARS-CoV-2 strain identified in coinfection with Influenza A (H1N1)pdm09.

On the other hand, phylogenetic analysis of viral receptor spike (S) sequence revealed our all five SARS-CoV-2 strain belonged to Omicron lineage BA 2.75 and had mutations in spike (S), ORF 1a/b, ORF 3a, 6 and 8 and E, M, and N protein (Fig. 7, Table 4). These five cases comprising one ILI and four SARI cases were not observed to have any mutational difference in structural and non-structural gene sequences (Table 4).

**Table 4:** Mutation pattern noticed in SARS-CoV-2 Omicron BA.2.75.

S. no.	SARS-CoV-2 proteins	Mutational sites
	Spike protein	T19I, L24S, I210V, V213G, G257S, G339H, S371F, S373P, S375F, T376A, D405N, R408S, K417N, N440K, G446S, N460K, S477N, T478K, E484A, Q498R, N501Y, Y505H, D614G, H655Y, N679K, P681H, N764K, D796Y, Q954H and N969K
	ORF1a	S135R, T842I, S1221L, G1307S, P1640S, L3027F, T3090I, L3201F, T3255I, P3395H and N4060S
	ORF1b	P314L, G662S, R1315C, I1566V and T2163I
	ORF3a	T223I
	E protein	T9I and T11A,
	M protein	Q19E and A63T,
	ORF6	D61L
	ORF8	S84L
	N protein	P13L, R203K, G204R and S413R

## Discussion

To the best of our knowledge, the present study would be the first of its nature from central India to report the screening of both Influenza and COVID-19 from a total of 633 cases of ILI (297 cases) and SARI (336 cases) to investigate the etiological speciation, genetic relatedness, mutational characterization and their plausible effect in protein structure and relevant function. ILI cases showed 20.9% prevalence of H1N1pdm09 while in SARI cases, it was found 23.2%. Among 140 H1N1pdm09 cases, five were found coinfected with SARS-CoV-2 to show 0.78% coinfection rate. These coinfection cases included four (04) SARI (1.19%) and one ILI (0.3%). On literature search, 0.04% coinfection rate was reported from western, northern and southern India^1^. Another recent pan India surveillance study has reported 0.09% coinfection rate in ILI and SARI cases^8^. H1N1pdm09 viruses identified in the ILI and SARI cases were belonged to clade 6B.1A.5a.2 and were genetically related to vaccine strains A/Perth/34/2020, A/Victoria/2570/2019 and A/Victoria/88/2020. SARS-CoV-2 from five of the cases belonged to Omicron lineage BA2.75.

H1N1pdm09 contain two glycoprotein, the HA and NA. HA act as receptor binding and fusion protein while NA ensues receptor destroying activity to facilitate release of progeny virions from infected cells, decoy receptors and prevents virus self-aggregation^18^. Both plays an important role in host tropism, pathogenesis, transmission and are prime targets of immune response. Thus genetic monitoring of HA and NA may help in understanding viral changes and their effect on host-virus parasite relationship. HA consist of two domains HA1 and HA2. HA1 representing globular head domain consist 327 aa (position 1–327) while the HA2 representing the stalk domain comprising 222 aa (position 328–548). Seasonal Influenza vaccine elicits antibody (Ab) response mainly against HA1 while chimeric HA based universal vaccine elicit Ab response against HA2^19^. Thus, there is all likely possibility that in the event of antigenic drift, the protective humoral immune response generated either by seasonal Influenza vaccine eliciting strain specific antibody against HA1 or chimeric universal Influenza vaccine inducing broadly cross reactive antibodies against HA2 could prove inefficient^19–23^. Any single aa substitution in HA1 and HA2 may result in antigenic drift. Therefore, identifying these mutations were of utmost significance.

In all HA sequences, among 24 pan signature mutations in both ILI and SARI, substitutions at positions S74R, S84N, D97N, N129D, K130N, N156K, L161I, S162N, K163Q, S164T, S183P, S185I, S203T, I216T, V250A, A256T, N260D were found in the head domain HA1 while in HA2 representing stalk region exhibited four mutations of E374K, E451K, E499 K and E506D. These mutations were reported to help the virus for immune escape and plausibly enhanced the adaptability of the virus for high rate of transmissibility^19^. The five antibody-recognizing sites (antigenic sites) identified on HA protein were Ca1 (residues 169–173, 206–208, 238–240) Ca2 (residues 140–145, 224–225), Cb (residues 74–79), Sa (aa residues 128–129, 156–160, 162–167) and Sb (aa residues 187–198). We had observed six nonsynomous substitution in antibody recognizing Sa sites namely S74R at Cb and N129D, N156K, S162N, K163Q, S164T. These substitution could be the reason of inefficient antibody response^19,24^. I216T substitution was observed in the receptor binding site (Fig. 5E) and could plausibly alter the adaptability of the virus for enhanced transmissibility efficiency of the Influenza A pdm09 strain to cause outbreaks. The noted mutational substitutions however not likely to alter the overall 3D tertiary structure of HA (Fig. 5A). Previous reports revealed the functional effects of these common mutations in virulence by destabilizing antibody binding site or alter its orientation to the incoming glycan ligand or trigger conformational changes on ligand binding^25–27^. H1N1pdm09 viral strains carrying N156K, N129D/K130N and S162N/K163Q/S164T mutations could lead to antigenic drift and alter the antibody response of the Influenza vaccine^19,28^. K130N has been suggested to cause decreased receptor binding avidity. SARI/ILI cases analysis of H1N1 mutation pattern revealed some interesting findings. HA novel mutations Y78H, T508K, S523T and G6D, Y7F, P212L, G339R specifically were observed in the H1N1pdm09 strains from SARI cases. Theses mutations could be the probable reason of enhanced virulence of the H1N1 virus enabling more clinical manifestations in SARI cases requiring further study to ascertain their role. Similarly, I96R and C538F observed only in ILI cases requiring further studies to check its specific role. On comparison with WHO recommended egg based vaccine strain Inf A/Victoria/2570/2019 and cell culture based vaccine strain Inf A/Wisconsin/588/2019, except P83S, mutations for HA and NA mentioned in Tables 2, 3 were not inherited in vaccine strains and thus needs to consider for future vaccine candidate specially for Indian population.

The phylogenetic analysis revealed an evolutionary pattern of H1N1pdm09 which was found congruent with its isolation period. Interestingly, majority of HA mutational pattern revealed to the pre-established clade 6B and some of the mutation belonged to circulating viruses have fallen into clade 6B.1A, defined by the aa substitutions S74R, S84N, S162N, S164T, I216T, and I295V. The additional aa changes that further characterized to clade 6B.1A.5a (5a) include the HA substitutions N129D, S183P, T185I and N260D. Clade 5a has further diverged into two major subclades: 5a.1, defined by aa substitutions D187A and Q189E in HA and 5a.2, defined by K130N, N156K, L161I, V250A and E506D. Subclade 5a.2 viruses collected after August 2021 have additional HA1 mutation of K54Q, A186T, Q189E, E224A, R259K and K308R. All these substitution specific to 5a.2 found in our sequences confirm the circulation of H1N1pdm09 of clade 6B.1A.5a.2 in Chhattisgarh. H1N1pdm09 viruses belonging to both subclades have circulated during 2021–2022 in different geographic locations.

NA on the other hand plays a key role in virion release from cells, decoy receptors and act as an important target for antiviral drugs and antibodies^18^. The NA amino acid sequences showed a total of twenty six pan signature mutations in both ILI and SARI cases. Among these twenty six pan mutation, NA was reported to have an 16 amino acid substitution (N248D, V106I, N369K, V241I, N44S, I106V, N200S, I321V, I34V/K432E, N386K, L40T, I314M and V13I/V264I/N270K) fixed in H1N1pdm09 virus population from 2009 Influenza pandemic to till 2015 to indicate their biological relevance^18^. These substitutions were affecting both antigenic and enzymatic properties with several mutations affecting multiple properties. K432E was reported to affect catalytic activity, substrate specificity and the pH optimum to alter antigenicity of NA to enable it to cleave sialosides at low pH during virus entry to enhance virus replication and transmission rate^18^. Earlier studies have reported the substitution of N386K and K432E in bringing antigenic drift in H1N1pdm09 by inhibiting H1N1pdm09 specific monoclonal antibodies^20,29^. Substitutions close to the active site were N200S, N248D, V241I and K432E and among these K432E had the largest effect on NA catalytic activity. V106I was also reported to increase low pH activity of NA and also affect Ca^2+^ dependent thermo-stability of NA. N369K increased the cleavage of fetuin containing α-2,3 linked sialic acid. Dual V241I and N369K mutation were reported to enhance the in-vitro activity and fitness of contemporary H1N1pdm09 viruses in ferrets^30^. V241I and N369K are the permissive mutation which were reported to be present in > 99.9% of circulating H1N1pdm09 virus to confer virus the robust fitness in acquiring NA H275Y Oseltamivir resistance mutation and correlated with enhanced surface expression and enzymatic activity of the NA protein^30^. Further, the substitution V264I and N270K were reported to increase the cleavage of α2,6 linked sialic acid^18^. N248D, N369K and N449D also reported to alter NA surface resulting in loss of binding by human monoclonal antibodies leading to immune escape of the virus^20^. The biggest change in antigenic relatedness in 2013 was observed with occurrence of K432E and N386K among which the later mutation causes loss of glycosylation site. Analysis of SARI and ILI cases in respect of NA mutations revealed the presence of S299A exclusively in SARI requiring further studies to unravel its role.

We have the opinion that the mutations observed in both HA and NA most probably be occurred due to selection pressure either elicited by vaccination or natural viral infection to help the virus in evading immune response. The enhanced mutation in HA and NA in SARI than ILI cases could be the reason of more severity associated with SARI cases. The HA and NA mutational pattern would further prove pivotal in helping researcher and pharmaceutical companies to prepare future vaccine/chemotherapeutic formulation.

This study has reported five co-infection cases (0.78%) of SARS-CoV-2 and H1N1pdm09 in one ILI and four SARI cases. One of these SARI cases has succumbed to infection showing the severity of the co infection and underline the importance of early screening of these patients. Earlier studies have also reported coinfection from various countries indicating its global existence and the likely public health threat^4,5,31^. An earlier study from Northern, Western and Southern India had reported 0.04% coinfection of SARS-CoV-2/Influenza but no mortality^1^. This shows virus had geographically restrictive evolutionary changes. The likely explanation for the coinfection could be the incubation period of Influenza virus of 1–4 days found similar to the incubation period of SARS-CoV-2 varies from 1 to 14 days. SARS-CoV-2 infects the nasopharynx to cause mostly initial mild upper respiratory tract infection followed with further dissemination to the lower respiratory tract to inflict severe lower respiratory tract infection. Influenza virus similarly replicates in the upper respiratory tract and also spread to lower respiratory tract causing complications like pneumonia. Thus, human influenza virus and SARS-CoV-2 since having similar initial clinical presentation especially to cause fever and cough, needs to be identified to institute appropriate therapeutic interventions and infection control measures. We have reported SARS-CoV-2 Omicron (BA.2.75) variant in one ILI and four SARI cases with no specific mutational presentation for these clinical conditions. The predominance of omicron BA.2.75 over other omicron (BA.1.1.529, BA.2, BA.2.38, BA.2.43, BA.2.56, BA.2.74, BA.2.76, BA.5.2 and BA.5.2.1) was reported earlier by us^11^. Among the total 37 mutation detected in spike gene, the signature mutations of T19I and V213G in the N-terminal domain (NTD), S373P, S375F, T376A, and D405N in receptor-binding domain (RBD), D614G, H655Y, N679K, and P681H at the furin cleavage site, N764K and D796K in fusion peptide, and Q954H and N969K in heptapeptide repeat sequence (HR)1 were observed. Various non-synonymous mutations in spike protein have been implicated in virus infectivity, transmissibility, pathogenicity, immunological bypass, decreased neutralizing ability of monoclonal antibodies, high risk of reinfection, treatment failure, and even Omicron diagnostic detection failure^11^.

### Limitation

The limitation of the study is unable to sequence all the 140 cases of H1N1 due to financial constraint. We also did not follow up diseases severity pattern in monoinfected or coinfected individual as this study primarily focused on molecular characterization of the mono and coinfected cases. Further we felt five co-infection cases were not sufficient to decide and define specific genetic and clinical correlation. The specific HA and NA mutations in ILI and SARI cases warrants further study to ascertain their pathogenic role. Nonetheless, the finding of the study has provided the first ever data from Central India and could act as the baseline information for surveillance and future studies to understand mutation pattern underwent by both H1N1pdm09 and SARS-CoV-2.

## Conclusion

The present study disclosed H1N1pdm09 strains circulating in Chhattisgarh, India belonged to 6B.1A.5a.2. The regionally acquired mutations encompassing novel Y78H, T508K, S523T and G6D, Y7F, P212L, G339R in HA and S299A in NA domains in SARI cases may be associated with more clinical severity than ILI cases. These mutations allow viral adaptability for local geographical sustainability resulting in an efficient human transmissibility. HA and NA novel mutational pattern of H1N1pdm09 may potentially raise the possibility of antigenic drift resulting in looming threat of immune escape of the virus from vaccine induced monoclonal antibody response. Thus to thwart any possible sporadic outbreak by the mutated virus, it is recommended to vaccine manufacturer to match vaccine virus with circulatory mutated virus for effective vaccine induced immune response. Coinfection cases and one mortality underlines the importance of susceptibility of SARI and ILI cases to COVID-19 infection requiring immediate treatment management to thwart possible mortality. WGS surveillance to monitor mutational changes help in initiating appropriate public health measure in advance before the mutational driven enhanced transmissibility and virulence result in more severe infection and mortality.

### Supplementary Information

Supplementary Information.

## Data Availability

The datasets generated, submitted and analysed during the current study are available in the NCBI database using the NCBI Accession No: OR043045-OR043356 for Influenza A (H1N1)pdm09 and OR016160-OR016164 for SARS-CoV-2.
