# Supplementary material for: Genomic characterization of Influenza A (H1N1)pdm09 and SARS-CoV-2 from Influenza Like Illness (ILI) and Severe Acute Respiratory Illness (SARI) cases reported between July–December, 2022
Source: Sci Rep. 2024 May 9;14:10660. doi: 10.1038/s41598-024-58993-w (PMC11081947; doi:10.1038/s41598-024-58993-w)
Supplement: Supplementary file 1 — Supplementary Information. [file 41598_2024_58993_MOESM1_ESM.docx]

**Supplementary Table 1:**

**Influenza A virus 312 sequences (39 samples) Submitted in NCBI 25.05.2023 (Release Date 30.05.2023)**

| **S. No.** | **Virus name** | **Collection date** | **District** | **Gender** | **Age** | **NCBI Accession ID** |
| --- | --- | --- | --- | --- | --- | --- |
|  | Influenza A virus (A/India/CG-AIIMSR-115/2022 (H1N1)pdm09) segment 1 polymerase PB2 (PB2) gene | 03-08-22 | Durg | Male | 62 | **OR043045** |
|  | Influenza A virus (A/India/CG-AIIMSR-115/2022 (H1N1)pdm09) segment 2 polymerase PB1 (PB1) gene and nonfunctional PB1-F2 protein (PB1-F2) gene | 03-08-22 | Durg | Male | 62 | **OR043046** |
|  | Influenza A virus (A/India/CG-AIIMSR-115/2022 (H1N1)pdm09) segment 3 polymerase PA (PA) gene | 03-08-22 | Durg | Male | 62 | **OR043047** |
|  | Influenza A virus (A/India/CG-AIIMSR-115/2022 (H1N1)pdm09) segment 4 hemagglutinin (HA) gene | 03-08-22 | Durg | Male | 62 | **OR043048** |
|  | Influenza A virus (A/India/CG-AIIMSR-115/2022 (H1N1)pdm09) segment 5 nucleocapsid protein (NP) gene | 03-08-22 | Durg | Male | 62 | **OR043049** |
|  | Influenza A virus (A/India/CG-AIIMSR-115/2022 (H1N1)pdm09) segment 6 neuraminidase (NA) gene | 03-08-22 | Durg | Male | 62 | **OR043050** |
|  | Influenza A virus (A/India/CG-AIIMSR-115/2022 (H1N1)pdm09) segment 7 matrix protein 2 (M2) and matrix protein 1 (M1) genes | 03-08-22 | Durg | Male | 62 | **OR043051** |
|  | Influenza A virus (A/India/CG-AIIMSR-115/2022 (H1N1)pdm09) segment 8 nuclear export protein (NEP) and nonstructural protein 1 (NS1) genes | 03-08-22 | Durg | Male | 62 | **OR043052** |
|  | Influenza A virus (A/India/CG-AIIMSR-136/2022 (H1N1)pdm09) segment 1 polymerase PB2 (PB2) gene | 08-08-22 | Raipur | Male | 50 | **OR043053** |
|  | Influenza A virus (A/India/CG-AIIMSR-136/2022 (H1N1)pdm09) segment 2 polymerase PB1 (PB1) gene and nonfunctional PB1-F2 protein (PB1-F2) gene | 08-08-22 | Raipur | Male | 50 | **OR043054** |
|  | Influenza A virus (A/India/CG-AIIMSR-136/2022 (H1N1)pdm09) segment 3 polymerase PA (PA) gene | 08-08-22 | Raipur | Male | 50 | **OR043055** |
|  | Influenza A virus (A/India/CG-AIIMSR-136/2022 (H1N1)pdm09) segment 4 hemagglutinin (HA) gene | 08-08-22 | Raipur | Male | 50 | **OR043056** |
|  | Influenza A virus (A/India/CG-AIIMSR-136/2022 (H1N1)pdm09) segment 5 nucleocapsid protein (NP) gene | 08-08-22 | Raipur | Male | 50 | **OR043057** |
|  | Influenza A virus (A/India/CG-AIIMSR-136/2022 (H1N1)pdm09) segment 6 neuraminidase (NA) gene | 08-08-22 | Raipur | Male | 50 | **OR043058** |
|  | Influenza A virus (A/India/CG-AIIMSR-136/2022 (H1N1)pdm09) segment 7 matrix protein 2 (M2) and matrix protein 1 (M1) genes | 08-08-22 | Raipur | Male | 50 | **OR043059** |
|  | Influenza A virus (A/India/CG-AIIMSR-136/2022 (H1N1)pdm09) segment 8 nuclear export protein (NEP) and nonstructural protein 1 (NS1) genes | 08-08-22 | Raipur | Male | 50 | **OR043060** |
|  | Influenza A virus (A/India/CG-AIIMSR-155/2022 (H1N1)pdm09) segment 1 polymerase PB2 (PB2) gene | 11-08-22 | Raigarh | Female | 52 | **OR043061** |
|  | Influenza A virus (A/India/CG-AIIMSR-155/2022 (H1N1)pdm09) segment 2 polymerase PB1 (PB1) gene and nonfunctional PB1-F2 protein (PB1-F2) gene | 11-08-22 | Raigarh | Female | 52 | **OR043062** |
|  | Influenza A virus (A/India/CG-AIIMSR-155/2022 (H1N1)pdm09) segment 3 polymerase PA (PA) gene | 11-08-22 | Raigarh | Female | 52 | **OR043063** |
|  | Influenza A virus (A/India/CG-AIIMSR-155/2022 (H1N1)pdm09) segment 4 hemagglutinin (HA) gene | 11-08-22 | Raigarh | Female | 52 | **OR043064** |
|  | Influenza A virus (A/India/CG-AIIMSR-155/2022 (H1N1)pdm09) segment 5 nucleocapsid protein (NP) gene | 11-08-22 | Raigarh | Female | 52 | **OR043065** |
|  | Influenza A virus (A/India/CG-AIIMSR-155/2022 (H1N1)pdm09) segment 6 neuraminidase (NA) gene | 11-08-22 | Raigarh | Female | 52 | **OR043066** |
|  | Influenza A virus (A/India/CG-AIIMSR-155/2022 (H1N1)pdm09) segment 7 matrix protein 2 (M2) and matrix protein 1 (M1) genes | 11-08-22 | Raigarh | Female | 52 | **OR043067** |
|  | Influenza A virus (A/India/CG-AIIMSR-155/2022 (H1N1)pdm09) segment 8 nuclear export protein (NEP) and nonstructural protein 1 (NS1) genes | 11-08-22 | Raigarh | Female | 52 | **OR043068** |
|  | Influenza A virus (A/India/CG-AIIMSR-156/2022 (H1N1)pdm09) segment 1 polymerase PB2 (PB2) gene | 11-08-22 | Raigarh | Female | 52 | **OR043069** |
|  | Influenza A virus (A/India/CG-AIIMSR-156/2022 (H1N1)pdm09) segment 2 polymerase PB1 (PB1) gene and nonfunctional PB1-F2 protein (PB1-F2) gene | 11-08-22 | Raigarh | Female | 52 | **OR043070** |
|  | Influenza A virus (A/India/CG-AIIMSR-156/2022 (H1N1)pdm09) segment 3 polymerase PA (PA) gene | 11-08-22 | Raigarh | Female | 52 | **OR043071** |
|  | Influenza A virus (A/India/CG-AIIMSR-156/2022 (H1N1)pdm09) segment 4 hemagglutinin (HA) gene | 11-08-22 | Raigarh | Female | 52 | **OR043072** |
|  | Influenza A virus (A/India/CG-AIIMSR-156/2022 (H1N1)pdm09) segment 5 nucleocapsid protein (NP) gene | 11-08-22 | Raigarh | Female | 52 | **OR043073** |
|  | Influenza A virus (A/India/CG-AIIMSR-156/2022 (H1N1)pdm09) segment 6 neuraminidase (NA) gene | 11-08-22 | Raigarh | Female | 52 | **OR043074** |
|  | Influenza A virus (A/India/CG-AIIMSR-156/2022 (H1N1)pdm09) segment 7 matrix protein 2 (M2) and matrix protein 1 (M1) genes | 11-08-22 | Raigarh | Female | 52 | **OR043075** |
|  | Influenza A virus (A/India/CG-AIIMSR-156/2022 (H1N1)pdm09) segment 8 nuclear export protein (NEP) and nonstructural protein 1 (NS1) genes | 11-08-22 | Raigarh | Female | 52 | **OR043076** |
|  | Influenza A virus (A/India/CG-AIIMSR-165/2022 (H1N1)pdm09) segment 1 polymerase PB2 (PB2) gene | 13-08-22 | Durg | Male | 81 | **OR043077** |
|  | Influenza A virus (A/India/CG-AIIMSR-165/2022 (H1N1)pdm09) segment 2 polymerase PB1 (PB1) gene and nonfunctional PB1-F2 protein (PB1-F2) gene | 13-08-22 | Durg | Male | 81 | **OR043078** |
|  | Influenza A virus (A/India/CG-AIIMSR-165/2022 (H1N1)pdm09) segment 3 polymerase PA (PA) gene | 13-08-22 | Durg | Male | 81 | **OR043079** |
|  | Influenza A virus (A/India/CG-AIIMSR-165/2022 (H1N1)pdm09) segment 4 hemagglutinin (HA) gene | 13-08-22 | Durg | Male | 81 | **OR043080** |
|  | Influenza A virus (A/India/CG-AIIMSR-165/2022 (H1N1)pdm09) segment 5 nucleocapsid protein (NP) gene | 13-08-22 | Durg | Male | 81 | **OR043081** |
|  | Influenza A virus (A/India/CG-AIIMSR-165/2022 (H1N1)pdm09) segment 6 neuraminidase (NA) gene | 13-08-22 | Durg | Male | 81 | **OR043082** |
|  | Influenza A virus (A/India/CG-AIIMSR-165/2022 (H1N1)pdm09) segment 7 matrix protein 2 (M2) and matrix protein 1 (M1) genes | 13-08-22 | Durg | Male | 81 | **OR043083** |
|  | Influenza A virus (A/India/CG-AIIMSR-165/2022 (H1N1)pdm09) segment 8 nuclear export protein (NEP) and nonstructural protein 1 (NS1) genes | 13-08-22 | Durg | Male | 81 | **OR043084** |
|  | Influenza A virus (A/India/CG-AIIMSR-190/2022 (H1N1)pdm09) segment 1 polymerase PB2 (PB2) gene | 16-08-22 | Raipur | Male | 7 m | **OR043085** |
|  | Influenza A virus (A/India/CG-AIIMSR-190/2022 (H1N1)pdm09) segment 2 polymerase PB1 (PB1) gene and nonfunctional PB1-F2 protein (PB1-F2) gene | 16-08-22 | Raipur | Male | 7 m | **OR043086** |
|  | Influenza A virus (A/India/CG-AIIMSR-190/2022 (H1N1)pdm09) segment 3 polymerase PA (PA) gene | 16-08-22 | Raipur | Male | 7 m | **OR043087** |
|  | Influenza A virus (A/India/CG-AIIMSR-190/2022 (H1N1)pdm09) segment 4 hemagglutinin (HA) gene | 16-08-22 | Raipur | Male | 7 m | **OR043088** |
|  | Influenza A virus (A/India/CG-AIIMSR-190/2022 (H1N1)pdm09) segment 5 nucleocapsid protein (NP) gene | 16-08-22 | Raipur | Male | 7 m | **OR043089** |
|  | Influenza A virus (A/India/CG-AIIMSR-190/2022 (H1N1)pdm09) segment 6 neuraminidase (NA) gene | 16-08-22 | Raipur | Male | 7 m | **OR043090** |
|  | Influenza A virus (A/India/CG-AIIMSR-190/2022 (H1N1)pdm09) segment 7 matrix protein 2 (M2) and matrix protein 1 (M1) genes | 16-08-22 | Raipur | Male | 7 m | **OR043091** |
|  | Influenza A virus (A/India/CG-AIIMSR-190/2022 (H1N1)pdm09) segment 8 nuclear export protein (NEP) and nonstructural protein 1 (NS1) genes | 16-08-22 | Raipur | Male | 7 m | **OR043092** |
|  | Influenza A virus (A/India/CG-AIIMSR-202/2022 (H1N1)pdm09) segment 1 polymerase PB2 (PB2) gene | 20-08-22 | Raipur | Female | 56 | **OR043093** |
|  | Influenza A virus (A/India/CG-AIIMSR-202/2022 (H1N1)pdm09) segment 2 polymerase PB1 (PB1) gene and nonfunctional PB1-F2 protein (PB1-F2) gene | 20-08-22 | Raipur | Female | 56 | **OR043094** |
|  | Influenza A virus (A/India/CG-AIIMSR-202/2022 (H1N1)pdm09) segment 3 polymerase PA (PA) gene | 20-08-22 | Raipur | Female | 56 | **OR043095** |
|  | Influenza A virus (A/India/CG-AIIMSR-202/2022 (H1N1)pdm09) segment 4 hemagglutinin (HA) gene | 20-08-22 | Raipur | Female | 56 | **OR043096** |
|  | Influenza A virus (A/India/CG-AIIMSR-202/2022 (H1N1)pdm09) segment 5 nucleocapsid protein (NP) gene | 20-08-22 | Raipur | Female | 56 | **OR043097** |
|  | Influenza A virus (A/India/CG-AIIMSR-202/2022 (H1N1)pdm09) segment 6 neuraminidase (NA) gene | 20-08-22 | Raipur | Female | 56 | **OR043098** |
|  | Influenza A virus (A/India/CG-AIIMSR-202/2022 (H1N1)pdm09) segment 7 matrix protein 2 (M2) and matrix protein 1 (M1) genes | 20-08-22 | Raipur | Female | 56 | **OR043099** |
|  | Influenza A virus (A/India/CG-AIIMSR-202/2022 (H1N1)pdm09) segment 8 nuclear export protein (NEP) and nonstructural protein 1 (NS1) genes | 20-08-22 | Raipur | Female | 56 | **OR043100** |
|  | Influenza A virus (A/India/CG-AIIMSR-210/2022 (H1N1)pdm09) segment 1 polymerase PB2 (PB2) gene | 20-08-22 | Raipur | Female | 39 | **OR043101** |
|  | Influenza A virus (A/India/CG-AIIMSR-210/2022 (H1N1)pdm09) segment 2 polymerase PB1 (PB1) gene and nonfunctional PB1-F2 protein (PB1-F2) gene | 20-08-22 | Raipur | Female | 39 | **OR043102** |
|  | Influenza A virus (A/India/CG-AIIMSR-210/2022 (H1N1)pdm09) segment 3 polymerase PA (PA) gene | 20-08-22 | Raipur | Female | 39 | **OR043103** |
|  | Influenza A virus (A/India/CG-AIIMSR-210/2022 (H1N1)pdm09) segment 4 hemagglutinin (HA) gene | 20-08-22 | Raipur | Female | 39 | **OR043104** |
|  | Influenza A virus (A/India/CG-AIIMSR-210/2022 (H1N1)pdm09) segment 5 nucleocapsid protein (NP) gene | 20-08-22 | Raipur | Female | 39 | **OR043105** |
|  | Influenza A virus (A/India/CG-AIIMSR-210/2022 (H1N1)pdm09) segment 6 neuraminidase (NA) gene | 20-08-22 | Raipur | Female | 39 | **OR043106** |
|  | Influenza A virus (A/India/CG-AIIMSR-210/2022 (H1N1)pdm09) segment 7 matrix protein 2 (M2) and matrix protein 1 (M1) genes | 20-08-22 | Raipur | Female | 39 | **OR043107** |
|  | Influenza A virus (A/India/CG-AIIMSR-210/2022 (H1N1)pdm09) segment 8 nuclear export protein (NEP) and nonstructural protein 1 (NS1) genes | 20-08-22 | Raipur | Female | 39 | **OR043108** |
|  | Influenza A virus (A/India/CG-AIIMSR-212/2022 (H1N1)pdm09) segment 1 polymerase PB2 (PB2) gene | 29-08-22 | Raipur | Female | 25 | **OR043109** |
|  | Influenza A virus (A/India/CG-AIIMSR-212/2022 (H1N1)pdm09) segment 2 polymerase PB1 (PB1) gene and nonfunctional PB1-F2 protein (PB1-F2) gene | 29-08-22 | Raipur | Female | 25 | **OR043110** |
|  | Influenza A virus (A/India/CG-AIIMSR-212/2022 (H1N1)pdm09) segment 3 polymerase PA (PA) gene | 29-08-22 | Raipur | Female | 25 | **OR043111** |
|  | Influenza A virus (A/India/CG-AIIMSR-212/2022 (H1N1)pdm09) segment 4 hemagglutinin (HA) gene | 29-08-22 | Raipur | Female | 25 | **OR043112** |
|  | Influenza A virus (A/India/CG-AIIMSR-212/2022 (H1N1)pdm09) segment 5 nucleocapsid protein (NP) gene | 29-08-22 | Raipur | Female | 25 | **OR043113** |
|  | Influenza A virus (A/India/CG-AIIMSR-212/2022 (H1N1)pdm09) segment 6 neuraminidase (NA) gene | 29-08-22 | Raipur | Female | 25 | **OR043114** |
|  | Influenza A virus (A/India/CG-AIIMSR-212/2022 (H1N1)pdm09) segment 7 matrix protein 2 (M2) and matrix protein 1 (M1) genes | 29-08-22 | Raipur | Female | 25 | **OR043115** |
|  | Influenza A virus (A/India/CG-AIIMSR-212/2022 (H1N1)pdm09) segment 8 nuclear export protein (NEP) and nonstructural protein 1 (NS1) genes | 29-08-22 | Raipur | Female | 25 | **OR043116** |
|  | Influenza A virus (A/India/CG-AIIMSR-259/2022 (H1N1)pdm09) segment 1 polymerase PB2 (PB2) gene | 27-08-22 | Raipur | Male | 49 | **OR043117** |
|  | Influenza A virus (A/India/CG-AIIMSR-259/2022 (H1N1)pdm09) segment 2 polymerase PB1 (PB1) gene and nonfunctional PB1-F2 protein (PB1-F2) gene | 27-08-22 | Raipur | Male | 49 | **OR043118** |
|  | Influenza A virus (A/India/CG-AIIMSR-259/2022 (H1N1)pdm09) segment 3 polymerase PA (PA) gene | 27-08-22 | Raipur | Male | 49 | **OR043119** |
|  | Influenza A virus (A/India/CG-AIIMSR-259/2022 (H1N1)pdm09) segment 4 hemagglutinin (HA) gene | 27-08-22 | Raipur | Male | 49 | **OR043120** |
|  | Influenza A virus (A/India/CG-AIIMSR-259/2022 (H1N1)pdm09) segment 5 nucleocapsid protein (NP) gene | 27-08-22 | Raipur | Male | 49 | **OR043121** |
|  | Influenza A virus (A/India/CG-AIIMSR-259/2022 (H1N1)pdm09) segment 6 neuraminidase (NA) gene | 27-08-22 | Raipur | Male | 49 | **OR043122** |
|  | Influenza A virus (A/India/CG-AIIMSR-259/2022 (H1N1)pdm09) segment 7 matrix protein 2 (M2) and matrix protein 1 (M1) genes | 27-08-22 | Raipur | Male | 49 | **OR043123** |
|  | Influenza A virus (A/India/CG-AIIMSR-259/2022 (H1N1)pdm09) segment 8 nuclear export protein (NEP) and nonstructural protein 1 (NS1) genes | 27-08-22 | Raipur | Male | 49 | **OR043124** |
|  | Influenza A virus (A/India/CG-AIIMSR-260/2022 (H1N1)pdm09) segment 1 polymerase PB2 (PB2) gene | 27-08-22 | Raipur | Female | 32 | **OR043125** |
|  | Influenza A virus (A/India/CG-AIIMSR-260/2022 (H1N1)pdm09) segment 2 polymerase PB1 (PB1) gene and nonfunctional PB1-F2 protein (PB1-F2) gene | 27-08-22 | Raipur | Female | 32 | **OR043126** |
|  | Influenza A virus (A/India/CG-AIIMSR-260/2022 (H1N1)pdm09) segment 3 polymerase PA (PA) gene | 27-08-22 | Raipur | Female | 32 | **OR043127** |
|  | Influenza A virus (A/India/CG-AIIMSR-260/2022 (H1N1)pdm09) segment 4 hemagglutinin (HA) gene | 27-08-22 | Raipur | Female | 32 | **OR043128** |
|  | Influenza A virus (A/India/CG-AIIMSR-260/2022 (H1N1)pdm09) segment 5 nucleocapsid protein (NP) gene | 27-08-22 | Raipur | Female | 32 | **OR043129** |
|  | Influenza A virus (A/India/CG-AIIMSR-260/2022 (H1N1)pdm09) segment 6 neuraminidase (NA) gene | 27-08-22 | Raipur | Female | 32 | **OR043130** |
|  | Influenza A virus (A/India/CG-AIIMSR-260/2022 (H1N1)pdm09) segment 7 matrix protein 2 (M2) and matrix protein 1 (M1) genes | 27-08-22 | Raipur | Female | 32 | **OR043131** |
|  | Influenza A virus (A/India/CG-AIIMSR-260/2022 (H1N1)pdm09) segment 8 nuclear export protein (NEP) and nonstructural protein 1 (NS1) genes | 27-08-22 | Raipur | Female | 32 | **OR043132** |
|  | Influenza A virus (A/India/CG-AIIMSR-266/2022 (H1N1)pdm09) segment 1 polymerase PB2 (PB2) gene | 27-08-22 | Raipur | Male | 17 | **OR043133** |
|  | Influenza A virus (A/India/CG-AIIMSR-266/2022 (H1N1)pdm09) segment 2 polymerase PB1 (PB1) gene and nonfunctional PB1-F2 protein (PB1-F2) gene | 27-08-22 | Raipur | Male | 17 | **OR043134** |
|  | Influenza A virus (A/India/CG-AIIMSR-266/2022 (H1N1)pdm09) segment 3 polymerase PA (PA) gene | 27-08-22 | Raipur | Male | 17 | **OR043135** |
|  | Influenza A virus (A/India/CG-AIIMSR-266/2022 (H1N1)pdm09) segment 4 hemagglutinin (HA) gene | 27-08-22 | Raipur | Male | 17 | **OR043136** |
|  | Influenza A virus (A/India/CG-AIIMSR-266/2022 (H1N1)pdm09) segment 5 nucleocapsid protein (NP) gene | 27-08-22 | Raipur | Male | 17 | **OR043137** |
|  | Influenza A virus (A/India/CG-AIIMSR-266/2022 (H1N1)pdm09) segment 6 neuraminidase (NA) gene | 27-08-22 | Raipur | Male | 17 | **OR043138** |
|  | Influenza A virus (A/India/CG-AIIMSR-266/2022 (H1N1)pdm09) segment 7 matrix protein 2 (M2) and matrix protein 1 (M1) genes | 27-08-22 | Raipur | Male | 17 | **OR043139** |
|  | Influenza A virus (A/India/CG-AIIMSR-266/2022 (H1N1)pdm09) segment 8 nuclear export protein (NEP) and nonstructural protein 1 (NS1) genes | 27-08-22 | Raipur | Male | 17 | **OR043140** |
|  | Influenza A virus (A/India/CG-AIIMSR-268/2022 (H1N1)pdm09) segment 1 polymerase PB2 (PB2) gene | 27-08-22 | Raipur | Male | 40 | **OR043141** |
|  | Influenza A virus (A/India/CG-AIIMSR-268/2022 (H1N1)pdm09) segment 2 polymerase PB1 (PB1) gene and nonfunctional PB1-F2 protein (PB1-F2) gene | 27-08-22 | Raipur | Male | 40 | **OR043142** |
|  | Influenza A virus (A/India/CG-AIIMSR-268/2022 (H1N1)pdm09) segment 3 polymerase PA (PA) gene | 27-08-22 | Raipur | Male | 40 | **OR043143** |
|  | Influenza A virus (A/India/CG-AIIMSR-268/2022 (H1N1)pdm09) segment 4 hemagglutinin (HA) gene | 27-08-22 | Raipur | Male | 40 | **OR043144** |
|  | Influenza A virus (A/India/CG-AIIMSR-268/2022 (H1N1)pdm09) segment 5 nucleocapsid protein (NP) gene | 27-08-22 | Raipur | Male | 40 | **OR043145** |
|  | Influenza A virus (A/India/CG-AIIMSR-268/2022 (H1N1)pdm09) segment 6 neuraminidase (NA) gene | 27-08-22 | Raipur | Male | 40 | **OR043146** |
|  | Influenza A virus (A/India/CG-AIIMSR-268/2022 (H1N1)pdm09) segment 7 matrix protein 2 (M2) and matrix protein 1 (M1) genes | 27-08-22 | Raipur | Male | 40 | **OR043147** |
|  | Influenza A virus (A/India/CG-AIIMSR-268/2022 (H1N1)pdm09) segment 8 nuclear export protein (NEP) and nonstructural protein 1 (NS1) genes | 27-08-22 | Raipur | Male | 40 | **OR043148** |
|  | Influenza A virus (A/India/CG-AIIMSR-275/2022 (H1N1)pdm09) segment 1 polymerase PB2 (PB2) gene | 29-08-22 | Raipur | Male | 49 | **OR043149** |
|  | Influenza A virus (A/India/CG-AIIMSR-275/2022 (H1N1)pdm09) segment 2 polymerase PB1 (PB1) gene and nonfunctional PB1-F2 protein (PB1-F2) gene | 29-08-22 | Raipur | Male | 49 | **OR043150** |
|  | Influenza A virus (A/India/CG-AIIMSR-275/2022 (H1N1)pdm09) segment 3 polymerase PA (PA) gene | 29-08-22 | Raipur | Male | 49 | **OR043151** |
|  | Influenza A virus (A/India/CG-AIIMSR-275/2022 (H1N1)pdm09) segment 4 hemagglutinin (HA) gene | 29-08-22 | Raipur | Male | 49 | **OR043152** |
|  | Influenza A virus (A/India/CG-AIIMSR-275/2022 (H1N1)pdm09) segment 5 nucleocapsid protein (NP) gene | 29-08-22 | Raipur | Male | 49 | **OR043153** |
|  | Influenza A virus (A/India/CG-AIIMSR-275/2022 (H1N1)pdm09) segment 6 neuraminidase (NA) gene | 29-08-22 | Raipur | Male | 49 | **OR043154** |
|  | Influenza A virus (A/India/CG-AIIMSR-275/2022 (H1N1)pdm09) segment 7 matrix protein 2 (M2) and matrix protein 1 (M1) genes | 29-08-22 | Raipur | Male | 49 | **OR043155** |
|  | Influenza A virus (A/India/CG-AIIMSR-275/2022 (H1N1)pdm09) segment 8 nuclear export protein (NEP) and nonstructural protein 1 (NS1) genes | 29-08-22 | Raipur | Male | 49 | **OR043156** |
|  | Influenza A virus (A/India/CG-AIIMSR-292/2022 (H1N1)pdm09) segment 1 polymerase PB2 (PB2) gene | 30-08-22 | Raipur | Male | 09 | **OR043157** |
|  | Influenza A virus (A/India/CG-AIIMSR-292/2022 (H1N1)pdm09) segment 2 polymerase PB1 (PB1) gene and nonfunctional PB1-F2 protein (PB1-F2) gene | 30-08-22 | Raipur | Male | 09 | **OR043158** |
|  | Influenza A virus (A/India/CG-AIIMSR-292/2022 (H1N1)pdm09) segment 3 polymerase PA (PA) gene | 30-08-22 | Raipur | Male | 09 | **OR043159** |
|  | Influenza A virus (A/India/CG-AIIMSR-292/2022 (H1N1)pdm09) segment 4 hemagglutinin (HA) gene | 30-08-22 | Raipur | Male | 09 | **OR043160** |
|  | Influenza A virus (A/India/CG-AIIMSR-292/2022 (H1N1)pdm09) segment 5 nucleocapsid protein (NP) gene | 30-08-22 | Raipur | Male | 09 | **OR043161** |
|  | Influenza A virus (A/India/CG-AIIMSR-292/2022 (H1N1)pdm09) segment 6 neuraminidase (NA) gene | 30-08-22 | Raipur | Male | 09 | **OR043162** |
|  | Influenza A virus (A/India/CG-AIIMSR-292/2022 (H1N1)pdm09) segment 7 matrix protein 2 (M2) and matrix protein 1 (M1) genes | 30-08-22 | Raipur | Male | 09 | **OR043163** |
|  | Influenza A virus (A/India/CG-AIIMSR-292/2022 (H1N1)pdm09) segment 8 nuclear export protein (NEP) and nonstructural protein 1 (NS1) genes | 30-08-22 | Raipur | Male | 09 | **OR043164** |
|  | Influenza A virus (A/India/CG-AIIMSR-295/2022 (H1N1)pdm09) segment 1 polymerase PB2 (PB2) gene | 30-08-22 | Durg | Male | 22 | **OR043165** |
|  | Influenza A virus (A/India/CG-AIIMSR-295/2022 (H1N1)pdm09) segment 2 polymerase PB1 (PB1) gene and nonfunctional PB1-F2 protein (PB1-F2) gene | 30-08-22 | Durg | Male | 22 | **OR043166** |
|  | Influenza A virus (A/India/CG-AIIMSR-295/2022 (H1N1)pdm09) segment 3 polymerase PA (PA) gene | 30-08-22 | Durg | Male | 22 | **OR043167** |
|  | Influenza A virus (A/India/CG-AIIMSR-295/2022 (H1N1)pdm09) segment 4 hemagglutinin (HA) gene | 30-08-22 | Durg | Male | 22 | **OR043168** |
|  | Influenza A virus (A/India/CG-AIIMSR-295/2022 (H1N1)pdm09) segment 5 nucleocapsid protein (NP) gene | 30-08-22 | Durg | Male | 22 | **OR043169** |
|  | Influenza A virus (A/India/CG-AIIMSR-295/2022 (H1N1)pdm09) segment 6 neuraminidase (NA) gene | 30-08-22 | Durg | Male | 22 | **OR043170** |
|  | Influenza A virus (A/India/CG-AIIMSR-295/2022 (H1N1)pdm09) segment 7 matrix protein 2 (M2) and matrix protein 1 (M1) genes | 30-08-22 | Durg | Male | 22 | **OR043171** |
|  | Influenza A virus (A/India/CG-AIIMSR-295/2022 (H1N1)pdm09) segment 8 nuclear export protein (NEP) and nonstructural protein 1 (NS1) genes | 30-08-22 | Durg | Male | 22 | **OR043172** |
|  | Influenza A virus (A/India/CG-AIIMSR-299/2022 (H1N1)pdm09) segment 1 polymerase PB2 (PB2) gene | 30-08-22 | Raipur | Female | 30 | **OR043173** |
|  | Influenza A virus (A/India/CG-AIIMSR-299/2022 (H1N1)pdm09) segment 2 polymerase PB1 (PB1) gene and nonfunctional PB1-F2 protein (PB1-F2) gene | 30-08-22 | Raipur | Female | 30 | **OR043174** |
|  | Influenza A virus (A/India/CG-AIIMSR-299/2022 (H1N1)pdm09) segment 3 polymerase PA (PA) gene | 30-08-22 | Raipur | Female | 30 | **OR043175** |
|  | Influenza A virus (A/India/CG-AIIMSR-299/2022 (H1N1)pdm09) segment 4 hemagglutinin (HA) gene | 30-08-22 | Raipur | Female | 30 | **OR043176** |
|  | Influenza A virus (A/India/CG-AIIMSR-299/2022 (H1N1)pdm09) segment 5 nucleocapsid protein (NP) gene | 30-08-22 | Raipur | Female | 30 | **OR043177** |
|  | Influenza A virus (A/India/CG-AIIMSR-299/2022 (H1N1)pdm09) segment 6 neuraminidase (NA) gene | 30-08-22 | Raipur | Female | 30 | **OR043178** |
|  | Influenza A virus (A/India/CG-AIIMSR-299/2022 (H1N1)pdm09) segment 7 matrix protein 2 (M2) and matrix protein 1 (M1) genes | 30-08-22 | Raipur | Female | 30 | **OR043179** |
|  | Influenza A virus (A/India/CG-AIIMSR-299/2022 (H1N1)pdm09) segment 8 nuclear export protein (NEP) and nonstructural protein 1 (NS1) genes | 30-08-22 | Raipur | Female | 30 | **OR043180** |
|  | Influenza A virus (A/India/CG-AIIMSR-348/2022 (H1N1)pdm09) segment 1 polymerase PB2 (PB2) gene | 05-09-22 | Raigarh | Male | 26 | **OR043181** |
|  | Influenza A virus (A/India/CG-AIIMSR-348/2022 (H1N1)pdm09) segment 2 polymerase PB1 (PB1) gene and nonfunctional PB1-F2 protein (PB1-F2) gene | 05-09-22 | Raigarh | Male | 26 | **OR043182** |
|  | Influenza A virus (A/India/CG-AIIMSR-348/2022 (H1N1)pdm09) segment 3 polymerase PA (PA) gene | 05-09-22 | Raigarh | Male | 26 | **OR043183** |
|  | Influenza A virus (A/India/CG-AIIMSR-348/2022 (H1N1)pdm09) segment 4 hemagglutinin (HA) gene | 05-09-22 | Raigarh | Male | 26 | **OR043184** |
|  | Influenza A virus (A/India/CG-AIIMSR-348/2022 (H1N1)pdm09) segment 5 nucleocapsid protein (NP) gene | 05-09-22 | Raigarh | Male | 26 | **OR043185** |
|  | Influenza A virus (A/India/CG-AIIMSR-348/2022 (H1N1)pdm09) segment 6 neuraminidase (NA) gene | 05-09-22 | Raigarh | Male | 26 | **OR043186** |
|  | Influenza A virus (A/India/CG-AIIMSR-348/2022 (H1N1)pdm09) segment 7 matrix protein 2 (M2) and matrix protein 1 (M1) genes | 05-09-22 | Raigarh | Male | 26 | **OR043187** |
|  | Influenza A virus (A/India/CG-AIIMSR-348/2022 (H1N1)pdm09) segment 8 nuclear export protein (NEP) and nonstructural protein 1 (NS1) genes | 05-09-22 | Raigarh | Male | 26 | **OR043188** |
|  | Influenza A virus (A/India/CG-AIIMSR-393/2022 (H1N1)pdm09) segment 1 polymerase PB2 (PB2) gene | 06-09-22 | Raipur | Female | 58 | **OR043189** |
|  | Influenza A virus (A/India/CG-AIIMSR-393/2022 (H1N1)pdm09) segment 2 polymerase PB1 (PB1) gene and nonfunctional PB1-F2 protein (PB1-F2) gene | 06-09-22 | Raipur | Female | 58 | **OR043190** |
|  | Influenza A virus (A/India/CG-AIIMSR-393/2022 (H1N1)pdm09) segment 3 polymerase PA (PA) gene | 06-09-22 | Raipur | Female | 58 | **OR043191** |
|  | Influenza A virus (A/India/CG-AIIMSR-393/2022 (H1N1)pdm09) segment 4 hemagglutinin (HA) gene | 06-09-22 | Raipur | Female | 58 | **OR043192** |
|  | Influenza A virus (A/India/CG-AIIMSR-393/2022 (H1N1)pdm09) segment 5 nucleocapsid protein (NP) gene | 06-09-22 | Raipur | Female | 58 | **OR043193** |
|  | Influenza A virus (A/India/CG-AIIMSR-393/2022 (H1N1)pdm09) segment 6 neuraminidase (NA) gene | 06-09-22 | Raipur | Female | 58 | **OR043194** |
|  | Influenza A virus (A/India/CG-AIIMSR-393/2022 (H1N1)pdm09) segment 7 matrix protein 2 (M2) and matrix protein 1 (M1) genes | 06-09-22 | Raipur | Female | 58 | **OR043195** |
|  | Influenza A virus (A/India/CG-AIIMSR-393/2022 (H1N1)pdm09) segment 8 nuclear export protein (NEP) and nonstructural protein 1 (NS1) genes | 06-09-22 | Raipur | Female | 58 | **OR043196** |
|  | Influenza A virus (A/India/CG-AIIMSR-406/2022 (H1N1)pdm09) segment 1 polymerase PB2 (PB2) gene | 07-09-22 | Raipur | Male | NIL | **OR043197** |
|  | Influenza A virus (A/India/CG-AIIMSR-406/2022 (H1N1)pdm09) segment 2 polymerase PB1 (PB1) gene and nonfunctional PB1-F2 protein (PB1-F2) gene | 07-09-22 | Raipur | Male | NIL | **OR043198** |
|  | Influenza A virus (A/India/CG-AIIMSR-406/2022 (H1N1)pdm09) segment 3 polymerase PA (PA) gene | 07-09-22 | Raipur | Male | NIL | **OR043199** |
|  | Influenza A virus (A/India/CG-AIIMSR-406/2022 (H1N1)pdm09) segment 4 hemagglutinin (HA) gene | 07-09-22 | Raipur | Male | NIL | **OR043200** |
|  | Influenza A virus (A/India/CG-AIIMSR-406/2022 (H1N1)pdm09) segment 5 nucleocapsid protein (NP) gene | 07-09-22 | Raipur | Male | NIL | **OR043201** |
|  | Influenza A virus (A/India/CG-AIIMSR-406/2022 (H1N1)pdm09) segment 6 neuraminidase (NA) gene | 07-09-22 | Raipur | Male | NIL | **OR043202** |
|  | Influenza A virus (A/India/CG-AIIMSR-406/2022 (H1N1)pdm09) segment 7 matrix protein 2 (M2) and matrix protein 1 (M1) genes | 07-09-22 | Raipur | Male | NIL | **OR043203** |
|  | Influenza A virus (A/India/CG-AIIMSR-406/2022 (H1N1)pdm09) segment 8 nuclear export protein (NEP) and nonstructural protein 1 (NS1) genes | 07-09-22 | Raipur | Male | NIL | **OR043204** |
|  | Influenza A virus (A/India/CG-AIIMSR-414/2022 (H1N1)pdm09) segment 1 polymerase PB2 (PB2) gene | 08-09-22 | Raipur | Male | 29 | **OR043205** |
|  | Influenza A virus (A/India/CG-AIIMSR-414/2022 (H1N1)pdm09) segment 2 polymerase PB1 (PB1) gene and nonfunctional PB1-F2 protein (PB1-F2) gene | 08-09-22 | Raipur | Male | 29 | **OR043206** |
|  | Influenza A virus (A/India/CG-AIIMSR-414/2022 (H1N1)pdm09) segment 3 polymerase PA (PA) gene | 08-09-22 | Raipur | Male | 29 | **OR043207** |
|  | Influenza A virus (A/India/CG-AIIMSR-414/2022 (H1N1)pdm09) segment 4 hemagglutinin (HA) gene | 08-09-22 | Raipur | Male | 29 | **OR043208** |
|  | Influenza A virus (A/India/CG-AIIMSR-414/2022 (H1N1)pdm09) segment 5 nucleocapsid protein (NP) gene | 08-09-22 | Raipur | Male | 29 | **OR043209** |
|  | Influenza A virus (A/India/CG-AIIMSR-414/2022 (H1N1)pdm09) segment 6 neuraminidase (NA) gene | 08-09-22 | Raipur | Male | 29 | **OR043210** |
|  | Influenza A virus (A/India/CG-AIIMSR-414/2022 (H1N1)pdm09) segment 7 matrix protein 2 (M2) and matrix protein 1 (M1) genes | 08-09-22 | Raipur | Male | 29 | **OR043211** |
|  | Influenza A virus (A/India/CG-AIIMSR-414/2022 (H1N1)pdm09) segment 8 nuclear export protein (NEP) and nonstructural protein 1 (NS1) genes | 08-09-22 | Raipur | Male | 29 | **OR043212** |
|  | Influenza A virus (A/India/CG-AIIMSR-429/2022 (H1N1)pdm09) segment 1 polymerase PB2 (PB2) gene | 08-09-22 | Raipur | Male | 30 | **OR043213** |
|  | Influenza A virus (A/India/CG-AIIMSR-429/2022 (H1N1)pdm09) segment 2 polymerase PB1 (PB1) gene and nonfunctional PB1-F2 protein (PB1-F2) gene | 08-09-22 | Raipur | Male | 30 | **OR043214** |
|  | Influenza A virus (A/India/CG-AIIMSR-429/2022 (H1N1)pdm09) segment 3 polymerase PA (PA) gene | 08-09-22 | Raipur | Male | 30 | **OR043215** |
|  | Influenza A virus (A/India/CG-AIIMSR-429/2022 (H1N1)pdm09) segment 4 hemagglutinin (HA) gene | 08-09-22 | Raipur | Male | 30 | **OR043216** |
|  | Influenza A virus (A/India/CG-AIIMSR-429/2022 (H1N1)pdm09) segment 5 nucleocapsid protein (NP) gene | 08-09-22 | Raipur | Male | 30 | **OR043217** |
|  | Influenza A virus (A/India/CG-AIIMSR-429/2022 (H1N1)pdm09) segment 6 neuraminidase (NA) gene | 08-09-22 | Raipur | Male | 30 | **OR043218** |
|  | Influenza A virus (A/India/CG-AIIMSR-429/2022 (H1N1)pdm09) segment 7 matrix protein 2 (M2) and matrix protein 1 (M1) genes | 08-09-22 | Raipur | Male | 30 | **OR043219** |
|  | Influenza A virus (A/India/CG-AIIMSR-429/2022 (H1N1)pdm09) segment 8 nuclear export protein (NEP) and nonstructural protein 1 (NS1) genes | 08-09-22 | Raipur | Male | 30 | **OR043220** |
|  | Influenza A virus (A/India/CG-AIIMSR-434/2022 (H1N1)pdm09) segment 1 polymerase PB2 (PB2) gene | 08-09-22 | Raipur | Female | 16 | **OR043221** |
|  | Influenza A virus (A/India/CG-AIIMSR-434/2022 (H1N1)pdm09) segment 2 polymerase PB1 (PB1) gene and nonfunctional PB1-F2 protein (PB1-F2) gene | 08-09-22 | Raipur | Female | 16 | **OR043222** |
|  | Influenza A virus (A/India/CG-AIIMSR-434/2022 (H1N1)pdm09) segment 3 polymerase PA (PA) gene | 08-09-22 | Raipur | Female | 16 | **OR043223** |
|  | Influenza A virus (A/India/CG-AIIMSR-434/2022 (H1N1)pdm09) segment 4 hemagglutinin (HA) gene | 08-09-22 | Raipur | Female | 16 | **OR043224** |
|  | Influenza A virus (A/India/CG-AIIMSR-434/2022 (H1N1)pdm09) segment 5 nucleocapsid protein (NP) gene | 08-09-22 | Raipur | Female | 16 | **OR043225** |
|  | Influenza A virus (A/India/CG-AIIMSR-434/2022 (H1N1)pdm09) segment 6 neuraminidase (NA) gene | 08-09-22 | Raipur | Female | 16 | **OR043226** |
|  | Influenza A virus (A/India/CG-AIIMSR-434/2022 (H1N1)pdm09) segment 7 matrix protein 2 (M2) and matrix protein 1 (M1) genes | 08-09-22 | Raipur | Female | 16 | **OR043227** |
|  | Influenza A virus (A/India/CG-AIIMSR-434/2022 (H1N1)pdm09) segment 8 nuclear export protein (NEP) and nonstructural protein 1 (NS1) genes | 08-09-22 | Raipur | Female | 16 | **OR043228** |
|  | Influenza A virus (A/India/CG-AIIMSR-439/2022 (H1N1)pdm09) segment 1 polymerase PB2 (PB2) gene | 09-09-22 | Raipur | Male | 55 | **OR043229** |
|  | Influenza A virus (A/India/CG-AIIMSR-439/2022 (H1N1)pdm09) segment 2 polymerase PB1 (PB1) gene and nonfunctional PB1-F2 protein (PB1-F2) gene | 09-09-22 | Raipur | Male | 55 | **OR043230** |
|  | Influenza A virus (A/India/CG-AIIMSR-439/2022 (H1N1)pdm09) segment 3 polymerase PA (PA) gene | 09-09-22 | Raipur | Male | 55 | **OR043231** |
|  | Influenza A virus (A/India/CG-AIIMSR-439/2022 (H1N1)pdm09) segment 4 hemagglutinin (HA) gene | 09-09-22 | Raipur | Male | 55 | **OR043232** |
|  | Influenza A virus (A/India/CG-AIIMSR-439/2022 (H1N1)pdm09) segment 5 nucleocapsid protein (NP) gene | 09-09-22 | Raipur | Male | 55 | **OR043233** |
|  | Influenza A virus (A/India/CG-AIIMSR-439/2022 (H1N1)pdm09) segment 6 neuraminidase (NA) gene | 09-09-22 | Raipur | Male | 55 | **OR043234** |
|  | Influenza A virus (A/India/CG-AIIMSR-439/2022 (H1N1)pdm09) segment 7 matrix protein 2 (M2) and matrix protein 1 (M1) genes | 09-09-22 | Raipur | Male | 55 | **OR043235** |
|  | Influenza A virus (A/India/CG-AIIMSR-439/2022 (H1N1)pdm09) segment 8 nuclear export protein (NEP) and nonstructural protein 1 (NS1) genes | 09-09-22 | Raipur | Male | 55 | **OR043236** |
|  | Influenza A virus (A/India/CG-AIIMSR-508/2022 (H1N1)pdm09) segment 1 polymerase PB2 (PB2) gene | 14-09-22 | Raipur | Female | 5 | **OR043237** |
|  | Influenza A virus (A/India/CG-AIIMSR-508/2022 (H1N1)pdm09) segment 2 polymerase PB1 (PB1) gene and nonfunctional PB1-F2 protein (PB1-F2) gene | 14-09-22 | Raipur | Female | 5 | **OR043238** |
|  | Influenza A virus (A/India/CG-AIIMSR-508/2022 (H1N1)pdm09) segment 3 polymerase PA (PA) gene | 14-09-22 | Raipur | Female | 5 | **OR043239** |
|  | Influenza A virus (A/India/CG-AIIMSR-508/2022 (H1N1)pdm09) segment 4 hemagglutinin (HA) gene | 14-09-22 | Raipur | Female | 5 | **OR043240** |
|  | Influenza A virus (A/India/CG-AIIMSR-508/2022 (H1N1)pdm09) segment 5 nucleocapsid protein (NP) gene | 14-09-22 | Raipur | Female | 5 | **OR043241** |
|  | Influenza A virus (A/India/CG-AIIMSR-508/2022 (H1N1)pdm09) segment 6 neuraminidase (NA) gene | 14-09-22 | Raipur | Female | 5 | **OR043242** |
|  | Influenza A virus (A/India/CG-AIIMSR-508/2022 (H1N1)pdm09) segment 7 matrix protein 2 (M2) and matrix protein 1 (M1) genes | 14-09-22 | Raipur | Female | 5 | **OR043243** |
|  | Influenza A virus (A/India/CG-AIIMSR-508/2022 (H1N1)pdm09) segment 8 nuclear export protein (NEP) and nonstructural protein 1 (NS1) genes | 14-09-22 | Raipur | Female | 5 | **OR043244** |
|  | Influenza A virus (A/India/CG-AIIMSR-513/2022 (H1N1)pdm09) segment 1 polymerase PB2 (PB2) gene | 14-09-22 | Raipur | Male | 28 | **OR043245** |
|  | Influenza A virus (A/India/CG-AIIMSR-513/2022 (H1N1)pdm09) segment 2 polymerase PB1 (PB1) gene and nonfunctional PB1-F2 protein (PB1-F2) gene | 14-09-22 | Raipur | Male | 28 | **OR043246** |
|  | Influenza A virus (A/India/CG-AIIMSR-513/2022 (H1N1)pdm09) segment 3 polymerase PA (PA) gene | 14-09-22 | Raipur | Male | 28 | **OR043247** |
|  | Influenza A virus (A/India/CG-AIIMSR-513/2022 (H1N1)pdm09) segment 4 hemagglutinin (HA) gene | 14-09-22 | Raipur | Male | 28 | **OR043248** |
|  | Influenza A virus (A/India/CG-AIIMSR-513/2022 (H1N1)pdm09) segment 5 nucleocapsid protein (NP) gene | 14-09-22 | Raipur | Male | 28 | **OR043249** |
|  | Influenza A virus (A/India/CG-AIIMSR-513/2022 (H1N1)pdm09) segment 6 neuraminidase (NA) gene | 14-09-22 | Raipur | Male | 28 | **OR043250** |
|  | Influenza A virus (A/India/CG-AIIMSR-513/2022 (H1N1)pdm09) segment 7 matrix protein 2 (M2) and matrix protein 1 (M1) genes | 14-09-22 | Raipur | Male | 28 | **OR043251** |
|  | Influenza A virus (A/India/CG-AIIMSR-513/2022 (H1N1)pdm09) segment 8 nuclear export protein (NEP) and nonstructural protein 1 (NS1) genes | 14-09-22 | Raipur | Male | 28 | **OR043252** |
|  | Influenza A virus (A/India/CG-AIIMSR-523/2022 (H1N1)pdm09) segment 1 polymerase PB2 (PB2) gene | 15-09-22 | Raipur | Female | 28 | **OR043253** |
|  | Influenza A virus (A/India/CG-AIIMSR-523/2022 (H1N1)pdm09) segment 2 polymerase PB1 (PB1) gene and nonfunctional PB1-F2 protein (PB1-F2) gene | 15-09-22 | Raipur | Female | 28 | **OR043254** |
|  | Influenza A virus (A/India/CG-AIIMSR-523/2022 (H1N1)pdm09) segment 3 polymerase PA (PA) gene | 15-09-22 | Raipur | Female | 28 | **OR043255** |
|  | Influenza A virus (A/India/CG-AIIMSR-523/2022 (H1N1)pdm09) segment 4 hemagglutinin (HA) gene | 15-09-22 | Raipur | Female | 28 | **OR043256** |
|  | Influenza A virus (A/India/CG-AIIMSR-523/2022 (H1N1)pdm09) segment 5 nucleocapsid protein (NP) gene | 15-09-22 | Raipur | Female | 28 | **OR043257** |
|  | Influenza A virus (A/India/CG-AIIMSR-523/2022 (H1N1)pdm09) segment 6 neuraminidase (NA) gene | 15-09-22 | Raipur | Female | 28 | **OR043258** |
|  | Influenza A virus (A/India/CG-AIIMSR-523/2022 (H1N1)pdm09) segment 7 matrix protein 2 (M2) and matrix protein 1 (M1) genes | 15-09-22 | Raipur | Female | 28 | **OR043259** |
|  | Influenza A virus (A/India/CG-AIIMSR-523/2022 (H1N1)pdm09) segment 8 nuclear export protein (NEP) and nonstructural protein 1 (NS1) genes | 15-09-22 | Raipur | Female | 28 | **OR043260** |
|  | Influenza A virus (A/India/CG-AIIMSR-530/2022 (H1N1)pdm09) segment 1 polymerase PB2 (PB2) gene | 15-09-22 | Raipur | Male | 45 | **OR043261** |
|  | Influenza A virus (A/India/CG-AIIMSR-530/2022 (H1N1)pdm09) segment 2 polymerase PB1 (PB1) gene and nonfunctional PB1-F2 protein (PB1-F2) gene | 15-09-22 | Raipur | Male | 45 | **OR043262** |
|  | Influenza A virus (A/India/CG-AIIMSR-530/2022 (H1N1)pdm09) segment 3 polymerase PA (PA) gene | 15-09-22 | Raipur | Male | 45 | **OR043263** |
|  | Influenza A virus (A/India/CG-AIIMSR-530/2022 (H1N1)pdm09) segment 4 hemagglutinin (HA) gene | 15-09-22 | Raipur | Male | 45 | **OR043264** |
|  | Influenza A virus (A/India/CG-AIIMSR-530/2022 (H1N1)pdm09) segment 5 nucleocapsid protein (NP) gene | 15-09-22 | Raipur | Male | 45 | **OR043265** |
|  | Influenza A virus (A/India/CG-AIIMSR-530/2022 (H1N1)pdm09) segment 6 neuraminidase (NA) gene | 15-09-22 | Raipur | Male | 45 | **OR043266** |
|  | Influenza A virus (A/India/CG-AIIMSR-530/2022 (H1N1)pdm09) segment 7 matrix protein 2 (M2) and matrix protein 1 (M1) genes | 15-09-22 | Raipur | Male | 45 | **OR043267** |
|  | Influenza A virus (A/India/CG-AIIMSR-530/2022 (H1N1)pdm09) segment 8 nuclear export protein (NEP) and nonstructural protein 1 (NS1) genes | 15-09-22 | Raipur | Male | 45 | **OR043268** |
|  | Influenza A virus (A/India/CG-AIIMSR-550/2022 (H1N1)pdm09) segment 1 polymerase PB2 (PB2) gene | 17-09-22 | Raipur | Male | 28 | **OR043269** |
|  | Influenza A virus (A/India/CG-AIIMSR-550/2022 (H1N1)pdm09) segment 2 polymerase PB1 (PB1) gene and nonfunctional PB1-F2 protein (PB1-F2) gene | 17-09-22 | Raipur | Male | 28 | **OR043270** |
|  | Influenza A virus (A/India/CG-AIIMSR-550/2022 (H1N1)pdm09) segment 3 polymerase PA (PA) gene | 17-09-22 | Raipur | Male | 28 | **OR043271** |
|  | Influenza A virus (A/India/CG-AIIMSR-550/2022 (H1N1)pdm09) segment 4 hemagglutinin (HA) gene | 17-09-22 | Raipur | Male | 28 | **OR043272** |
|  | Influenza A virus (A/India/CG-AIIMSR-550/2022 (H1N1)pdm09) segment 5 nucleocapsid protein (NP) gene | 17-09-22 | Raipur | Male | 28 | **OR043273** |
|  | Influenza A virus (A/India/CG-AIIMSR-550/2022 (H1N1)pdm09) segment 6 neuraminidase (NA) gene | 17-09-22 | Raipur | Male | 28 | **OR043274** |
|  | Influenza A virus (A/India/CG-AIIMSR-550/2022 (H1N1)pdm09) segment 7 matrix protein 2 (M2) and matrix protein 1 (M1) genes | 17-09-22 | Raipur | Male | 28 | **OR043275** |
|  | Influenza A virus (A/India/CG-AIIMSR-550/2022 (H1N1)pdm09) segment 8 nuclear export protein (NEP) and nonstructural protein 1 (NS1) genes | 17-09-22 | Raipur | Male | 28 | **OR043276** |
|  | Influenza A virus (A/India/CG-AIIMSR-556/2022 (H1N1)pdm09) segment 1 polymerase PB2 (PB2) gene | 17-09-22 | Raipur | Male | 16 | **OR043277** |
|  | Influenza A virus (A/India/CG-AIIMSR-556/2022 (H1N1)pdm09) segment 2 polymerase PB1 (PB1) gene and nonfunctional PB1-F2 protein (PB1-F2) gene | 17-09-22 | Raipur | Male | 16 | **OR043278** |
|  | Influenza A virus (A/India/CG-AIIMSR-556/2022 (H1N1)pdm09) segment 3 polymerase PA (PA) gene | 17-09-22 | Raipur | Male | 16 | **OR043279** |
|  | Influenza A virus (A/India/CG-AIIMSR-556/2022 (H1N1)pdm09) segment 4 hemagglutinin (HA) gene | 17-09-22 | Raipur | Male | 16 | **OR043280** |
|  | Influenza A virus (A/India/CG-AIIMSR-556/2022 (H1N1)pdm09) segment 5 nucleocapsid protein (NP) gene | 17-09-22 | Raipur | Male | 16 | **OR043281** |
|  | Influenza A virus (A/India/CG-AIIMSR-556/2022 (H1N1)pdm09) segment 6 neuraminidase (NA) gene | 17-09-22 | Raipur | Male | 16 | **OR043282** |
|  | Influenza A virus (A/India/CG-AIIMSR-556/2022 (H1N1)pdm09) segment 7 matrix protein 2 (M2) and matrix protein 1 (M1) genes | 17-09-22 | Raipur | Male | 16 | **OR043283** |
|  | Influenza A virus (A/India/CG-AIIMSR-556/2022 (H1N1)pdm09) segment 8 nuclear export protein (NEP) and nonstructural protein 1 (NS1) genes | 17-09-22 | Raipur | Male | 16 | **OR043284** |
|  | Influenza A virus (A/India/CG-AIIMSR-557/2022 (H1N1)pdm09) segment 1 polymerase PB2 (PB2) gene | 18-09-22 | Raipur | Male | 26 | **OR043285** |
|  | Influenza A virus (A/India/CG-AIIMSR-557/2022 (H1N1)pdm09) segment 2 polymerase PB1 (PB1) gene and nonfunctional PB1-F2 protein (PB1-F2) gene | 18-09-22 | Raipur | Male | 26 | **OR043286** |
|  | Influenza A virus (A/India/CG-AIIMSR-557/2022 (H1N1)pdm09) segment 3 polymerase PA (PA) gene | 18-09-22 | Raipur | Male | 26 | **OR043287** |
|  | Influenza A virus (A/India/CG-AIIMSR-557/2022 (H1N1)pdm09) segment 4 hemagglutinin (HA) gene | 18-09-22 | Raipur | Male | 26 | **OR043288** |
|  | Influenza A virus (A/India/CG-AIIMSR-557/2022 (H1N1)pdm09) segment 5 nucleocapsid protein (NP) gene | 18-09-22 | Raipur | Male | 26 | **OR043289** |
|  | Influenza A virus (A/India/CG-AIIMSR-557/2022 (H1N1)pdm09) segment 6 neuraminidase (NA) gene | 18-09-22 | Raipur | Male | 26 | **OR043290** |
|  | Influenza A virus (A/India/CG-AIIMSR-557/2022 (H1N1)pdm09) segment 7 matrix protein 2 (M2) and matrix protein 1 (M1) genes | 18-09-22 | Raipur | Male | 26 | **OR043291** |
|  | Influenza A virus (A/India/CG-AIIMSR-557/2022 (H1N1)pdm09) segment 8 nuclear export protein (NEP) and nonstructural protein 1 (NS1) genes | 18-09-22 | Raipur | Male | 26 | **OR043292** |
|  | Influenza A virus (A/India/CG-AIIMSR-572/2022 (H1N1)pdm09) segment 1 polymerase PB2 (PB2) gene | 19-09-22 | Raipur | Male | 02 | **OR043293** |
|  | Influenza A virus (A/India/CG-AIIMSR-572/2022 (H1N1)pdm09) segment 2 polymerase PB1 (PB1) gene and nonfunctional PB1-F2 protein (PB1-F2) gene | 19-09-22 | Raipur | Male | 02 | **OR043294** |
|  | Influenza A virus (A/India/CG-AIIMSR-572/2022 (H1N1)pdm09) segment 3 polymerase PA (PA) gene | 19-09-22 | Raipur | Male | 02 | **OR043295** |
|  | Influenza A virus (A/India/CG-AIIMSR-572/2022 (H1N1)pdm09) segment 4 hemagglutinin (HA) gene | 19-09-22 | Raipur | Male | 02 | **OR043296** |
|  | Influenza A virus (A/India/CG-AIIMSR-572/2022 (H1N1)pdm09) segment 5 nucleocapsid protein (NP) gene | 19-09-22 | Raipur | Male | 02 | **OR043297** |
|  | Influenza A virus (A/India/CG-AIIMSR-572/2022 (H1N1)pdm09) segment 6 neuraminidase (NA) gene | 19-09-22 | Raipur | Male | 02 | **OR043298** |
|  | Influenza A virus (A/India/CG-AIIMSR-572/2022 (H1N1)pdm09) segment 7 matrix protein 2 (M2) and matrix protein 1 (M1) genes | 19-09-22 | Raipur | Male | 02 | **OR043299** |
|  | Influenza A virus (A/India/CG-AIIMSR-572/2022 (H1N1)pdm09) segment 8 nuclear export protein (NEP) and nonstructural protein 1 (NS1) genes | 19-09-22 | Raipur | Male | 02 | **OR043300** |
|  | Influenza A virus (A/India/CG-AIIMSR-579/2022 (H1N1)pdm09) segment 1 polymerase PB2 (PB2) gene | 19-09-22 | Raipur | Male | 60 | **OR043301** |
|  | Influenza A virus (A/India/CG-AIIMSR-579/2022 (H1N1)pdm09) segment 2 polymerase PB1 (PB1) gene and nonfunctional PB1-F2 protein (PB1-F2) gene | 19-09-22 | Raipur | Male | 60 | **OR043302** |
|  | Influenza A virus (A/India/CG-AIIMSR-579/2022 (H1N1)pdm09) segment 3 polymerase PA (PA) gene | 19-09-22 | Raipur | Male | 60 | **OR043303** |
|  | Influenza A virus (A/India/CG-AIIMSR-579/2022 (H1N1)pdm09) segment 4 hemagglutinin (HA) gene | 19-09-22 | Raipur | Male | 60 | **OR043304** |
|  | Influenza A virus (A/India/CG-AIIMSR-579/2022 (H1N1)pdm09) segment 5 nucleocapsid protein (NP) gene | 19-09-22 | Raipur | Male | 60 | **OR043305** |
|  | Influenza A virus (A/India/CG-AIIMSR-579/2022 (H1N1)pdm09) segment 6 neuraminidase (NA) gene | 19-09-22 | Raipur | Male | 60 | **OR043306** |
|  | Influenza A virus (A/India/CG-AIIMSR-579/2022 (H1N1)pdm09) segment 7 matrix protein 2 (M2) and matrix protein 1 (M1) genes | 19-09-22 | Raipur | Male | 60 | **OR043307** |
|  | Influenza A virus (A/India/CG-AIIMSR-579/2022 (H1N1)pdm09) segment 8 nuclear export protein (NEP) and nonstructural protein 1 (NS1) genes | 19-09-22 | Raipur | Male | 60 | **OR043308** |
|  | Influenza A virus (A/India/CG-AIIMSR-580/2022 (H1N1)pdm09) segment 1 polymerase PB2 (PB2) gene | 19-09-22 | Raipur | Male | 57 | **OR043309** |
|  | Influenza A virus (A/India/CG-AIIMSR-580/2022 (H1N1)pdm09) segment 2 polymerase PB1 (PB1) gene and nonfunctional PB1-F2 protein (PB1-F2) gene | 19-09-22 | Raipur | Male | 57 | **OR043310** |
|  | Influenza A virus (A/India/CG-AIIMSR-580/2022 (H1N1)pdm09) segment 3 polymerase PA (PA) gene | 19-09-22 | Raipur | Male | 57 | **OR043311** |
|  | Influenza A virus (A/India/CG-AIIMSR-580/2022 (H1N1)pdm09) segment 4 hemagglutinin (HA) gene | 19-09-22 | Raipur | Male | 57 | **OR043312** |
|  | Influenza A virus (A/India/CG-AIIMSR-580/2022 (H1N1)pdm09) segment 5 nucleocapsid protein (NP) gene | 19-09-22 | Raipur | Male | 57 | **OR043313** |
|  | Influenza A virus (A/India/CG-AIIMSR-580/2022 (H1N1)pdm09) segment 6 neuraminidase (NA) gene | 19-09-22 | Raipur | Male | 57 | **OR043314** |
|  | Influenza A virus (A/India/CG-AIIMSR-580/2022 (H1N1)pdm09) segment 7 matrix protein 2 (M2) and matrix protein 1 (M1) genes | 19-09-22 | Raipur | Male | 57 | **OR043315** |
|  | Influenza A virus (A/India/CG-AIIMSR-580/2022 (H1N1)pdm09) segment 8 nuclear export protein (NEP) and nonstructural protein 1 (NS1) genes | 19-09-22 | Raipur | Male | 57 | **OR043316** |
|  | Influenza A virus (A/India/CG-AIIMSR-596/2022 (H1N1)pdm09) segment 1 polymerase PB2 (PB2) gene | 20-09-22 | Raipur | Male | 32 | **OR043317** |
|  | Influenza A virus (A/India/CG-AIIMSR-596/2022 (H1N1)pdm09) segment 2 polymerase PB1 (PB1) gene and nonfunctional PB1-F2 protein (PB1-F2) gene | 20-09-22 | Raipur | Male | 32 | **OR043318** |
|  | Influenza A virus (A/India/CG-AIIMSR-596/2022 (H1N1)pdm09) segment 3 polymerase PA (PA) gene | 20-09-22 | Raipur | Male | 32 | **OR043319** |
|  | Influenza A virus (A/India/CG-AIIMSR-596/2022 (H1N1)pdm09) segment 4 hemagglutinin (HA) gene | 20-09-22 | Raipur | Male | 32 | **OR043320** |
|  | Influenza A virus (A/India/CG-AIIMSR-596/2022 (H1N1)pdm09) segment 5 nucleocapsid protein (NP) gene | 20-09-22 | Raipur | Male | 32 | **OR043321** |
|  | Influenza A virus (A/India/CG-AIIMSR-596/2022 (H1N1)pdm09) segment 6 neuraminidase (NA) gene | 20-09-22 | Raipur | Male | 32 | **OR043322** |
|  | Influenza A virus (A/India/CG-AIIMSR-596/2022 (H1N1)pdm09) segment 7 matrix protein 2 (M2) and matrix protein 1 (M1) genes | 20-09-22 | Raipur | Male | 32 | **OR043323** |
|  | Influenza A virus (A/India/CG-AIIMSR-596/2022 (H1N1)pdm09) segment 8 nuclear export protein (NEP) and nonstructural protein 1 (NS1) genes | 20-09-22 | Raipur | Male | 32 | **OR043324** |
|  | Influenza A virus (A/India/CG-AIIMSR-598/2022 (H1N1)pdm09) segment 1 polymerase PB2 (PB2) gene | 20-09-22 | Raipur | Male | 43 | **OR043325** |
|  | Influenza A virus (A/India/CG-AIIMSR-598/2022 (H1N1)pdm09) segment 2 polymerase PB1 (PB1) gene and nonfunctional PB1-F2 protein (PB1-F2) gene | 20-09-22 | Raipur | Male | 43 | **OR043326** |
|  | Influenza A virus (A/India/CG-AIIMSR-598/2022 (H1N1)pdm09) segment 3 polymerase PA (PA) gene | 20-09-22 | Raipur | Male | 43 | **OR043327** |
|  | Influenza A virus (A/India/CG-AIIMSR-598/2022 (H1N1)pdm09) segment 4 hemagglutinin (HA) gene | 20-09-22 | Raipur | Male | 43 | **OR043328** |
|  | Influenza A virus (A/India/CG-AIIMSR-598/2022 (H1N1)pdm09) segment 5 nucleocapsid protein (NP) gene | 20-09-22 | Raipur | Male | 43 | **OR043329** |
|  | Influenza A virus (A/India/CG-AIIMSR-598/2022 (H1N1)pdm09) segment 6 neuraminidase (NA) gene | 20-09-22 | Raipur | Male | 43 | **OR043330** |
|  | Influenza A virus (A/India/CG-AIIMSR-598/2022 (H1N1)pdm09) segment 7 matrix protein 2 (M2) and matrix protein 1 (M1) genes | 20-09-22 | Raipur | Male | 43 | **OR043331** |
|  | Influenza A virus (A/India/CG-AIIMSR-598/2022 (H1N1)pdm09) segment 8 nuclear export protein (NEP) and nonstructural protein 1 (NS1) genes | 20-09-22 | Raipur | Male | 43 | **OR043332** |
|  | Influenza A virus (A/India/CG-AIIMSR-605/2022 (H1N1)pdm09) segment 1 polymerase PB2 (PB2) gene | 21-09-22 | Raipur | Female | 01 | **OR043333** |
|  | Influenza A virus (A/India/CG-AIIMSR-605/2022 (H1N1)pdm09) segment 2 polymerase PB1 (PB1) gene and nonfunctional PB1-F2 protein (PB1-F2) gene | 21-09-22 | Raipur | Female | 01 | **OR043334** |
|  | Influenza A virus (A/India/CG-AIIMSR-605/2022 (H1N1)pdm09) segment 3 polymerase PA (PA) gene | 21-09-22 | Raipur | Female | 01 | **OR043335** |
|  | Influenza A virus (A/India/CG-AIIMSR-605/2022 (H1N1)pdm09) segment 4 hemagglutinin (HA) gene | 21-09-22 | Raipur | Female | 01 | **OR043336** |
|  | Influenza A virus (A/India/CG-AIIMSR-605/2022 (H1N1)pdm09) segment 5 nucleocapsid protein (NP) gene | 21-09-22 | Raipur | Female | 01 | **OR043337** |
|  | Influenza A virus (A/India/CG-AIIMSR-605/2022 (H1N1)pdm09) segment 6 neuraminidase (NA) gene | 21-09-22 | Raipur | Female | 01 | **OR043338** |
|  | Influenza A virus (A/India/CG-AIIMSR-605/2022 (H1N1)pdm09) segment 7 matrix protein 2 (M2) and matrix protein 1 (M1) genes | 21-09-22 | Raipur | Female | 01 | **OR043339** |
|  | Influenza A virus (A/India/CG-AIIMSR-605/2022 (H1N1)pdm09) segment 8 nuclear export protein (NEP) and nonstructural protein 1 (NS1) genes | 21-09-22 | Raipur | Female | 01 | **OR043340** |
|  | Influenza A virus (A/India/CG-AIIMSR-611/2022 (H1N1)pdm09) segment 1 polymerase PB2 (PB2) gene | 21-09-22 | Raipur | Male | 23 | **OR043341** |
|  | Influenza A virus (A/India/CG-AIIMSR-611/2022 (H1N1)pdm09) segment 2 polymerase PB1 (PB1) gene and nonfunctional PB1-F2 protein (PB1-F2) gene | 21-09-22 | Raipur | Male | 23 | **OR043342** |
|  | Influenza A virus (A/India/CG-AIIMSR-611/2022 (H1N1)pdm09) segment 3 polymerase PA (PA) gene | 21-09-22 | Raipur | Male | 23 | **OR043343** |
|  | Influenza A virus (A/India/CG-AIIMSR-611/2022 (H1N1)pdm09) segment 4 hemagglutinin (HA) gene | 21-09-22 | Raipur | Male | 23 | **OR043344** |
|  | Influenza A virus (A/India/CG-AIIMSR-611/2022 (H1N1)pdm09) segment 5 nucleocapsid protein (NP) gene | 21-09-22 | Raipur | Male | 23 | **OR043345** |
|  | Influenza A virus (A/India/CG-AIIMSR-611/2022 (H1N1)pdm09) segment 6 neuraminidase (NA) gene | 21-09-22 | Raipur | Male | 23 | **OR043346** |
|  | Influenza A virus (A/India/CG-AIIMSR-611/2022 (H1N1)pdm09) segment 7 matrix protein 2 (M2) and matrix protein 1 (M1) genes | 21-09-22 | Raipur | Male | 23 | **OR043347** |
|  | Influenza A virus (A/India/CG-AIIMSR-611/2022 (H1N1)pdm09) segment 8 nuclear export protein (NEP) and nonstructural protein 1 (NS1) genes | 21-09-22 | Raipur | Male | 23 | **OR043348** |
|  | Influenza A virus (A/India/CG-AIIMSR-637/2022 (H1N1)pdm09) segment 1 polymerase PB2 (PB2) gene | 24-09-22 | Raipur | Female | 35 | **OR043349** |
|  | Influenza A virus (A/India/CG-AIIMSR-637/2022 (H1N1)pdm09) segment 2 polymerase PB1 (PB1) gene and nonfunctional PB1-F2 protein (PB1-F2) gene | 24-09-22 | Raipur | Female | 35 | **OR043350** |
|  | Influenza A virus (A/India/CG-AIIMSR-637/2022 (H1N1)pdm09) segment 3 polymerase PA (PA) gene | 24-09-22 | Raipur | Female | 35 | **OR043351** |
|  | Influenza A virus (A/India/CG-AIIMSR-637/2022 (H1N1)pdm09) segment 4 hemagglutinin (HA) gene | 24-09-22 | Raipur | Female | 35 | **OR043352** |
|  | Influenza A virus (A/India/CG-AIIMSR-637/2022 (H1N1)pdm09) segment 5 nucleocapsid protein (NP) gene | 24-09-22 | Raipur | Female | 35 | **OR043353** |
|  | Influenza A virus (A/India/CG-AIIMSR-637/2022 (H1N1)pdm09) segment 6 neuraminidase (NA) gene | 24-09-22 | Raipur | Female | 35 | **OR043354** |
|  | Influenza A virus (A/India/CG-AIIMSR-637/2022 (H1N1)pdm09) segment 7 matrix protein 2 (M2) and matrix protein 1 (M1) genes | 24-09-22 | Raipur | Female | 35 | **OR043355** |
|  | Influenza A virus (A/India/CG-AIIMSR-637/2022 (H1N1)pdm09) segment 8 nuclear export protein (NEP) and nonstructural protein 1 (NS1) genes | 24-09-22 | Raipur | Female | 35 | **OR043356** |

**SARS-CoV-2 Co-infection with Influenza A virus 05 sequences Submitted in NCBI 20.05.2023 (Release Date 20.05.2023)**

| **S. No.** | **Virus name** | **Collection date** | **District** | **Gender** | **Age** | **NCBI Accession ID** |
| --- | --- | --- | --- | --- | --- | --- |
|  | SARS-CoV-2/human/IND/CG-VRDL-AIIMS-268/2022 | 27-08-22 | Raipur | Male | 40 | **OR016160** |
|  | SARS-CoV-2/human/IND/CG-VRDL-AIIMS-295/2022 | 30-08-22 | Durg | Male | 22 | **OR016161** |
|  | SARS-CoV-2/human/IND/CG-VRDL-AIIMS-266/2022 | 27-08-22 | Raipur | Male | 17 | **OR016162** |
|  | SARS-CoV-2/human/IND/CG-VRDL-AIIMS-292/2022 | 30-08-22 | Raipur | Male | 09 | **OR016163** |
|  | SARS-CoV-2/human/IND/CG-VRDL-AIIMS-248/2022 | 05-09-22 | Raigarh | Male | 26 | **OR016164** |
